# Catalytic Properties of Zirconocene-Based Systems in 1-Hexene Oligomerization and Structure of Metal Hydride Reaction Centers

**DOI:** 10.3390/molecules28062420

**Published:** 2023-03-07

**Authors:** Lyudmila V. Parfenova, Pavel V. Kovyazin, Almira Kh. Bikmeeva, Eldar R. Palatov, Pavel V. Ivchenko, Ilya E. Nifant’ev, Leonard M. Khalilov

**Affiliations:** 1 Institute of Petrochemistry and Catalysis, Ufa Federal Research Center, Russian Academy of Sciences, Prosp. Oktyabrya, 141, 450075 Ufa, Russia; 2 Department of Chemistry, Lomonosov Moscow State University, 1-3 Leninskiye Gory, 119991 Moscow, Russia; 3 A.V. Topchiev Institute of Petrochemical Synthesis, Russian Academy of Sciences, Leninsky Prosp. 29, 119991 Moscow, Russia

**Keywords:** zirconocenes, metal hydrides, methylaluminoxane, perfluorophenyl borates, alkene dimerization, oligomerization, nuclear magnetic resonance

## Abstract

Despite large-scale investigations of homogeneous single-site metallocene catalysts and systems based on them, there are still unsolved problems related to the control of their activity and chemo- and stereoselectivity. A solution to these problems is required to develop efficient methods for the synthesis of practically useful products of alkene transformations, such as dimers, oligomers, and polymers. Here we studied the catalytic activity of structurally diverse zirconocenes (L_2_ZrCl_2_, L = Cp, C_5_Me_5_, Ind, L_2_ = Me_2_CCp_2_, Me_2_SiCp_2_, Me_2_C_2_Cp_2_, *rac*-Me_2_CInd_2_, *rac*-H_4_C_2_Ind_2_, BIPh(Ind)_2_, H_4_C_2_[THInd]_2_), and co-catalysts activating the system, namely HAlBu^i^_2_, MMAO-12, and (Ph_3_C)[B(C_6_F_5_)_4_], at low activator/Zr ratios in a 1-hexene oligomerization reaction. The influence of catalyst structure and system composition on the alkene conversion, the type of products, and the reaction stereoselectivity were investigated. The composition of hydride intermediates formed in the L_2_ZrCl_2_–HAlBu^i^_2_–activator system (L_2_ = ansa-Me_2_CCp_2_, Ind) was studied by NMR spectroscopy. Participation of the bis-zirconium hydride complex as the precursor of catalytically active sites of the alkene dimerization reaction was shown.

## 1. Introduction

Catalytic systems based on metallocenes, organoaluminum compounds (OACs), and activators are well known and have been studied for almost 70 years [1]. A large amount of research is related to the possibility of the efficient synthesis of practically important products: alkene dimers and oligomers, as well as polymers widely known as polyalphaolefins (PAO) [2,3,4,5,6,7]. The use of metallocene complexes in these reactions made it possible to transfer the classical Ziegler–Natta catalysis from a heterogeneous to a homogeneous medium. Hence, highly active and stereoselective single-site catalysts for the polymerization of unsaturated compounds became more controllable, and a detailed study of the reaction mechanism became possible. The high activity and chemo- and stereoselectivity of metallocene complexes are due to their structural features. They provide the most efficient stabilization of the electronic and steric environment of transition metal atoms owing to the high energy of metal–ligand bonds and allow variation of the electrophilicity and geometry of catalytically active sites due to broad possibilities of π-ligand modification. It has been shown that the electronic and steric features of the ligand, the nature of the activator, and the reaction conditions [8,9,10,11,12,13,14,15] determine the productivity and selectivity of catalytic systems.

There is a long-standing interest in the mechanisms of action of homogeneous single-site catalysts, which are constantly being improved and supplemented by new facts [16,17,18,19,20,21,22]. One of the trends in this field is the investigation of the role of hydride intermediates in alkene transformation reactions, especially in dimerization and oligomerization [6,7,23,24,25,26,27,28,29,30,31], the mechanism of which remains an open question. 

Our interest in the structural features and reactivity of group 4B metal hydrides is due to the recent data on the alkene transformation reactions catalyzed by zirconocene dichloride Cp_2_ZrCl_2_ or zirconocene dihydride [Cp_2_ZrH_2_]_2_ and OACs in the presence of aluminum- or boron-containing activators [32,33,34,35]. The formation of the bimetallic Zr,Zr-hydride intermediate in these systems was established, and its ability to serve as the precursor of the specific and selective catalytically active sites for alkene dimerization was demonstrated. 

The present work is aimed at the study of the catalytic activity of structurally diverse zirconocenes (10 examples of cyclopentadienyl and indenyl complexes) in the presence of activators, namely HAlBu^i^_2_, MMAO-12, and (Ph_3_C)[B(C_6_F_5_)_4_], at low activator/Zr ratios in 1-hexene oligomerization. The influence of catalyst structure and system composition on the alkene conversion and reaction chemo- and stereoselectivity was investigated. The structure of hydride intermediates formed in the L_2_ZrCl_2_–HAlBu^i^_2_–activator system (L_2_ = *ansa*-Me_2_CCp_2_, Ind) was studied by means of NMR spectroscopy.

## 2. Results and Discussion

### 2.1. Catalytic Effect of L_2_ZrCl_2_–HAlBu^i^_2_–Activator Systems in 1-Hexene Oligomerization

**Scheme 1:**
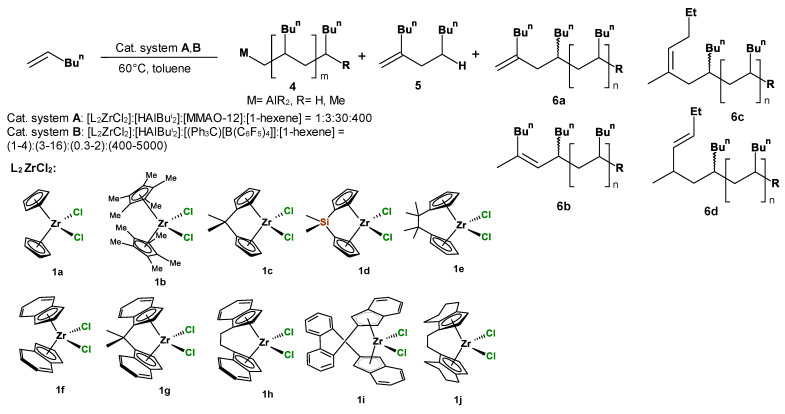
Alkene transformations in the presence of L_2_ZrCl_2_–HAlBu^i^_2_–activator catalytic systems.

We studied the catalytic effect of complexes **1a**–**j** in the oligomerization reaction in the presence of HAlBu^i^_2_ and the activators MMAO-12 (modified methylaluminoxane) and [Ph_3_C][B(C_6_F_5_)_4_]. We used di(isobutyl)alane HAlBu^i^_2_ as a co-catalyst similar to AlBu^i^_3_ [6,7,26,27,28,30] to initiate the formation of zirconocene hydrides, which act as catalytically active sites in these reactions [29,30,33,34,35]. The reactant ratios were chosen on the basis of our earlier studies [33,34,35]. During the reaction under the selected conditions, the conversion of 1-hexene was approximately 99% in 1–3 h in most of the experiments. A decrease in the alkene conversion down to 85% was observed in the reaction catalyzed by complex **1e** in the presence of MMAO-12. 1-Hexene dimer (**5**) and various oligomers (**6**) were formed as the major reaction products, which attests to predominant chain termination via *β*-H elimination mechanism [36] (Scheme 1, Figures S1 and S2). The presence of oligomers with various double bond positions may be due to the migration of zirconium cation upon the formation of allyl complexes [28,37]. Small amounts of M–C bonded oligomers **4** were generally observed in systems containing MMAO-12. In the same systems, oligomers with a methyl starting group were found. It should be noted that the application of other OACs (AlBu^i^_3_, AlEt_3_, or AlMe_3_) in the reaction provided the identical nature of the dimerization and oligomerization products (see, for example, Refs. [26,27,28,31,33,34]). The origin of hydride intermediates in these systems is a result of the *β*-H elimination process in isobutyl and ethyl groups, as well as in branched alkyl chains bonded with Zr atoms [36].

It was shown that in the system based on zirconocene dichloride and [Ph_3_C][B(C_6_F_5_)_4_] reacting in toluene, the best result was achieved at a lower content of activator [Ph_3_C][B(C_6_F_5_)_4_] relative to the Zr complex. Thus, Cp_2_ZrCl_2_ catalyzes the oligomerization reaction at the [Zr]:[B] ratio of 1:(0.3–1). When [Zr]:[B] = 1:0.3, the catalytic system was more selective towards dimerization (Table 1, entries 1, 4). An increase in the [Ph_3_C][B(C_6_F_5_)_4_] concentration in the range of 0.3–1 equiv. resulted in a higher proportion of heavier oligomers with *n* = 1–5. Simultaneously, the selectivity of the reaction decreased, as follows from the GC-MS and NMR spectra of the products, representing a mixture of stereoisomers and isomers differing in the double bond position. The ^13^C NMR spectra of the oligomers formed under these conditions exhibited signals for the internal double bond at δ_C_ 124–131 ppm. When the reaction was carried out at the [Zr]:[B] ratio of 1:2, alkylation products, hexyl-substituted toluenes, were formed as the major products. An increase in the relative content of zirconocene and HAlBu^i^_2_ provided the formation of heavier products with bimodal distribution (Mw 5571 and 1578 Da), along with dimers and light oligomers (Table 1, entry 4). In the presence of MMAO-12, this system showed higher activity and selectivity to dimers (entry 5) [31].

In the presence of complexes **1c** and **1e** with hydrocarbon-bridged cyclopentadienyl ligands, dimer 5 was formed in >94% yield (entries 8, 10, and 14). The activity and chemoselectivity of complex **1e** appeared to be higher with activator [Ph_3_C][B(C_6_F_5_)_4_] (98% dimer) compared to MMAO-12 (78% dimer) (entry 15).

Significant differences in the action of activators were also found when the reaction was carried out in the presence of Me_2_Si-bound complex **1d** (entries 12, 13). For example, the use of MMAO-12 promoted the formation of dimerization product **5** in up to 80% yield, whereas the application of [Ph_3_C][B(C_6_F_5_)_4_] resulted in the predominant formation of atactic oligomers **6**, including heavy oligomers with Mw 6130.

Carrying out the reaction in the presence of bulky pentamethylcyclopentadienyl complex **1b** gave mainly oligomer **6,** whose mass distribution depended on the type of activator used (entries 6, 7). Under the action of [Ph_3_C][B(C_6_F_5_)_4_], short-chain oligomers with vinylidene end groups were formed predominantly (^13^C NMR data). The heavy fraction obtained in the presence of MMAO-12 contained oligomers with a bimodal distribution (M_w_ 5605 and 861 Da). Moreover, the number of heavy oligomers was much higher in this case than in the experiment with sterically non-hindered Cp_2_ZrCl_2_. This indicates a significant predominance of the chain propagation rate over the chain termination rate under these conditions, which may be due to the steric hindrance of the catalytically active sites preventing *β*-H elimination or chain transfer to the monomer or OAC. Apparently, the steric hindrance is generated by not only the pentamethyl-substituted ligand on Zr but also the bulky methylaluminoxane counterion. 

The appearance of indeneyl ligands (**1f**), in particular bridged ligands (**1g–j**), in the complexes also led to an increased yield of oligomeric products, both for MMAO-12 and for [Ph_3_C][B(C_6_F_5_)_4_] (entries 16–33). The reaction catalyzed by Me_2_CInd_2_ZrCl_2_ (**1g**) mainly afforded dimers and light oligomers with *n* = 1–4 (entries 23–25). Heavy oligomers were formed in the system based on *rac*-H_4_C_2_Ind_2_ZrCl_2_ (**1h**) and [Ph_3_C][B(C_6_F_5_)_4_] or MMAO-12 activator (entries 26, 27). Hydrogenation of the ligand benzene rings in complex **1h** and the transition to **1j** resulted in a considerable decrease in the catalytic system activity: an alkene conversion at the 90–99% level was achieved in 3 h (entries 31–33). Moreover, the oligomeric products obtained in these experiments had, as a rule, a multimodal distribution.

For the isolated higher hexene oligomers, the isotacticity in mmmm% was determined using ^13^C NMR spectroscopy [37,38,39,40]. The highest stereoselectivity was found for zirconocenes with indenyl ligands. For example, complexes **1f, 1g**, and **1h** showed isotacticities of 67, 93, and 71% mmmm, respectively (Table 1, entries 20, 23, and 26) (Figures S3 and S4). The stereoselectivity of the catalytic systems based on zirconocenes **1i** and **1j** was below 58% mmmm. It is noteworthy that the stereoselectivity of this reaction depended on the nature of the activator used. Under the action of complex **1f**, an oligomer with isotacticity of 67% was obtained in the presence of MMAO-12, while [Ph_3_C][B(C_6_F_5_)_4_] furnished an atactic product. The opposite trend was observed for complex **1g** with bound ligands: the highest stereoselectivity was attained in the presence of [Ph_3_C][B(C_6_F_5_)_4_]. The complex H_4_C_2_Ind_2_ZrCl_2_ (**1h**) provides the formation of isotactic oligohexenes (61–71% mmmm) regardless of the activator type. For complexes **1i** and **1j**, some increase in the stereoselectivity was found in the presence of MMAO-12. The observed dependence indicates a fine-tuning of the catalytically active sites in the homogeneous Ziegler–Natta type systems. Probably, the stereocontrol may be provided by not only the ligand on the transition metal atom (site control) or the growing chain (chain control) but also the activator occurring as a part of the active site and determining its structure and dynamics on an equal basis with other factors. As stated earlier [12], the complete separation of the cation–anion pair may increase the activity of the system but may lead to the loss of stereoselectivity. Strong cation–anion interactions could minimize the epimerization of the last inserted unit and thus increase the stereoselectivity, but at the same time, they cause a decrease in the activity by hampering the monomer insertion. 

Thus, it was shown that the dimerization pathway is implemented with the participation of Zr complexes with sterically non-hindered cyclopentadienyl ligands (L = Cp, *ansa*-Me_2_CCp_2_, *ansa*-(Me_2_C)_2_Cp_2_, and *ansa*-Me_2_SiCp_2_), whereas the oligomerization is determined by the action of complexes with bulky cyclopentadienyl (L = C_5_Me_5_, *rac*-H_4_C_2_[THInd]_2_) or electron-withdrawing indenyl (L = Ind, Me_2_CInd_2_, H_4_C_2_Ind_2_, BIPh(Ind)_2_) ligands. The difference between the behaviors of the complexes is primarily determined by the composition and reactivity of the intermediates. It was assumed that hydride bimetallic intermediates may act as the active sites of dimerization and oligomerization reactions [29,30,33,34,35]. Our previous NMR study of the reaction of Cp_2_ZrCl_2_ or [Cp_2_ZrH_2_]_2_ with OACs showed the existence of Zr,Zr-hydride complexes, which are activated by MMAO-12 or organoboron compound ([Ph_3_C][B(C_6_F_5_)_4_] or B(C_6_F_5_)_3_), being thus converted to active species responsible for dimer formation [32,33,34,35].

### 2.2. NMR Study of the L_2_ZrCl_2_–HAlBu^i^_2_–Activator Catalytic Systems 

In order to elucidate the effect of the ligand environment of the transition metal on the structure and reactivity of hydride intermediates, we studied systems consisting of zirconocenes with *ansa*-cyclopentadienyl and indenyl ligands (**1c** and **1f**), HAlBu^i^_2_, and MMAO-12 or the ionic activator [Ph_3_C][B(C_6_F_5_)_4_].

**Scheme 2:**
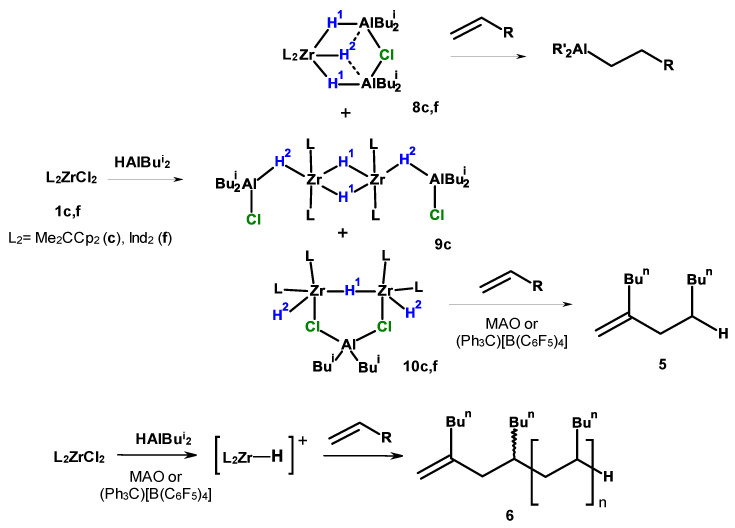
Reaction of Me_2_CCp_2_ZrCl_2_ (**1c**) or Ind_2_ZrCl_2_ (**1f**) with HAlBu^i^_2_, activators, and alkene.

It was found that the composition of the reaction mixture formed in the reaction of zirconocenes with HAlBu^i^_2_ largely depends on the starting reactant ratio. For example, the ^1^H NMR spectrum of the mixture of **1c** and HAlBu^i^_2_ taken in the molar ratio [Zr]:[Al] = 1:3 showed hydride signals at δ_H_ −1.44 (H^1^) and 1.29–1.43 ppm (H^2^). These signals were correlated with each other in the COSY HH experiment (at 250 K) (Figures S5 and S6, Table 2). The H^1^:H^Cp^:H^Cp^ ratio of signal intensities was 1:2:2. The NOESY spectrum exhibited opposite phase cross-peaks of these protons with Cp ring signals at δ_H_ 6.11 and 5.19 ppm, and a signal for the methyl group of the isopropylidene bridge at δ_H_ 1.03 ppm (Figure S7). At 298 K, the hydride signals were significantly broadened, and peaks corresponding to the chemical exchange between H^1^, H^2^, and HAlBu^i^_2_ monomers and oligomers appeared in the NOESY spectra (Figure S8). Considering these results and literature data for similar molecules with other π-ligands [41,42,43,44], we assigned the observed signals to the trihydride complex **8c** as the most probable structure (Scheme 2, Table 2). Moreover, the C_2v_ symmetry of the complex was also in line with the observed NMR pattern.

Under conditions of HAlBu^i^_2_ deficiency ([Zr]:[Al] = 1:1.7), the spectrum exhibited high-field signals at δ_H_ −1.10 (H^1^) and −0.81 (H^2^) ppm, correlated with each other in the COSY HH spectrum, in addition to the signals of unreacted complex **1c** (Figures S9 and S10). The low-field region of the spectrum contained signals of four non-equivalent protons of the cyclopentadienyl ligand at δ_H_ 6.28, 6.23, 5.66, and 5.37 ppm. The observed decrease in the symmetry of the structure, the H^1^:H^2^:H^Cp^:H^Cp^:H^Cp^:H^Cp^ intensity ratio of 1:1:2:2:2, and literature data [42,44] make it possible to identify the structure as **9c**. Additionally, the ^1^H NMR spectrum showed a low-intensity triplet at δ_H_ −3.58 ppm, which was correlated with a doublet at δ_H_ −1.34 ppm in the COSY HH spectra (Figure S10). The spin–spin coupling constants *^2^J* = 17.9 Hz for these signals are typical of the bis-zirconium structures [Cp_2_ZrH_2_∙Cp_2_ZrHCl∙ClAlR_2_], which we detected earlier [32,33,34,35]. Therefore, these resonance lines were attributed to complex **10c**.

**Figure 1:**
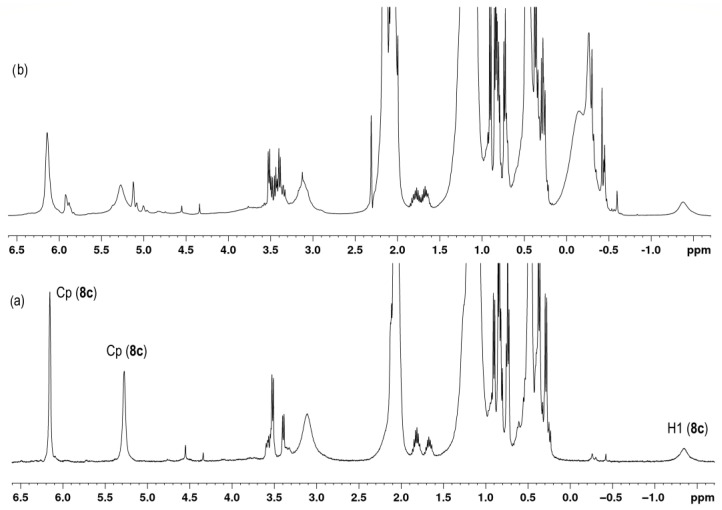
^1^H NMR spectrum of the Me_2_CCp_2_ZrCl_2_ (**1c**)−HAlBu^i^_2_−MMAO-12 system in C_7_D_8_ (298 K): (**a**) [Zr]:[Al]:[Al_MAO_] = 1:8:0; (**b**) [Zr]:[Al]:[Al_MAO_] = 1:8:11.

The addition of MMAO-12 to the mixture obtained in the reaction of Me_2_CCp_2_ZrCl_2_ and HAlBu^i^_2_ in a ratio of 1:(3–8) and containing the trihydride intermediate **8c** induced broadening of both hydride signals and cyclopentadienyl ring signals of complex **8c** (Figure 1 and Figure S13), which may indicate its association with methylaluminoxane. According to theoretical data, zirconium hydride complexes have a high affinity for this activator and are able to produce stable adducts [45]. In addition, it is known that methylaluminoxane tends to absorb hydrides, which exchange with methyl groups to provide H-substituted MAO (methylaluminoxane) [46]. This was also confirmed in our study. The NOESY spectra of a mixture of MMAO-12 and HAlBu^i^_2_ showed a negative effect for methyl groups (−0.14 ppm), isobutyl groups (0.56 and 1.29 ppm), and H-Al(MAO) (3.26–4.11 ppm) (Figures S11 and S12), indicating that these groups belong to the macromolecule. It also follows from the NOESY spectra that the association of **8c** with MAO preserves the possibility of hydride exchange with H-Al(MAO) (Figure S14).

Under conditions of HAlBu^i^_2_ deficiency, when complexes **9c** and **10c** existed in the catalytic system, the addition of MMAO-12 was accompanied by the appearance of a heavy fraction and by doubling of the triplet at δ_H_ −3.58 ppm belonging to **10c**, whereas the signals of complex **9c** remained virtually unchanged (Figures S15 and S16). The negative NOE indicated an increase in the molecular weight of the complex, which may be due to the formation of a stable **10c∙MAO** conjugate similar to the bis-cyclopentadienyl complex [33,35] (Figure S17).

The addition of [Ph_3_C][B(C_6_F_5_)_4_] to complex **8c** formed in the Me_2_CCp_2_ZrCl_2_–HAlBu^i^_2_ system (1:1) led to an equilibrium shift due to the reaction of HAlBu^i^_2_ with the organoboron reagent, resulting in the recovery of the original zirconocene dichloride and the appearance of complexes **9c** and **10c** (Figures S18 and S19). The NMR signals of complex **9c** disappeared over time.

**Figure 2:**
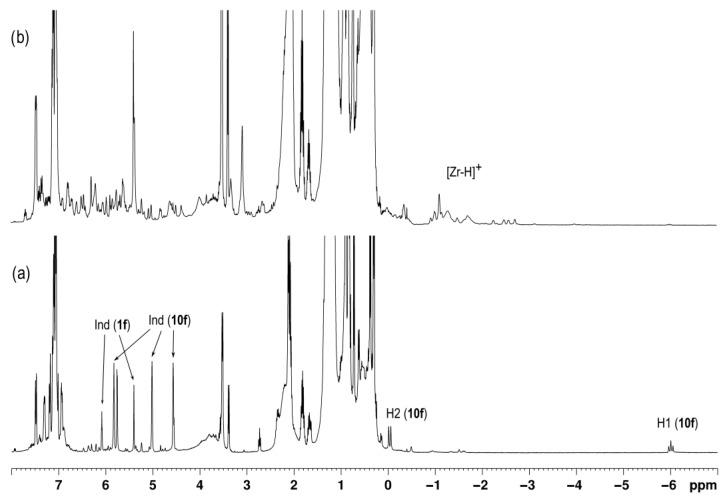
^1^H NMR spectrum of the Ind_2_ZrCl_2_ (**1f**)−HAlBu^i^_2_−[Ph_3_C][B(C_6_F_5_)_4_] system in C_7_D_8_ (298 K): (**a**) [Zr]:[Al]:[B] = 1:3:0.2; (**b**) [Zr]:[Al]:[B] = 1:8:0.2.

In the reaction of Ind_2_ZrCl_2_ (**1f**) with HAlBu^i^_2_, taken in a ratio of [Zr]:[Al] = 1:(3–8), complex **8f** was formed [43]. The complex was characterized by the presence of broadened high-field signals of hydride atoms in the ^1^H NMR spectra at room temperature, which indicated the participation of the molecule in exchange processes (Figures S21 and S22). The addition of the ionic activator [Ph_3_C][B(C_6_F_5_)_4_] to **8f**, obtained in the **1f**-HAlBu^i^_2_ system (1:3), provided complex **10f** and initial **1f** by analogy with the ansa-bis-cyclopentadienyl complex (Figure 2 and Figures S23 and S24, Table 2). Moreover, the spectra exhibited a set of indenyl proton signals at **δ_H_** 4.2–7.8 ppm and hydride atom signals at **δ_H_** −3–0 ppm, which can be tentatively assigned to the cationic hydride species. Their content increased as the concentration of HAlBu^i^_2_ increased in the system (Figure 2 and Figure S25).

As in the case of the cyclopentadienyl complex **8c**, the addition of MMAO-12 to **8f**, obtained in the reaction of **1f** with an excess of HAlBu^i^_2_ (1:3), gave the associate **10f**∙MAO, which was characterized by hydride signals at –6.11 and 0.05 ppm in the ^1^H NMR spectra (Figures S26 and S27). An increase in the content of HAlBu^i^_2_ to the ratio [Zr]:[Al] = 1:8 in the **1f**-HAlBu^i^_2_-MMAO-12 ([Ph_3_C][B(C_6_F_5_)_4_]) systems contributed to an increase the intensity of the ^1^H NMR signals corresponding probably to the cationic species [L_2_ZrH]^+^ (Figure 2b and Figures S25 and S28). It should be noted that under conditions of a large excess of HAlBu^i^_2_ (1:8), the complex **8f** did not provide associates with methylalumoxane similar to **8c**, which followed from the permanence of the shape and width of NMR spectral lines with the appearance of the activator.

**Figure 3:**
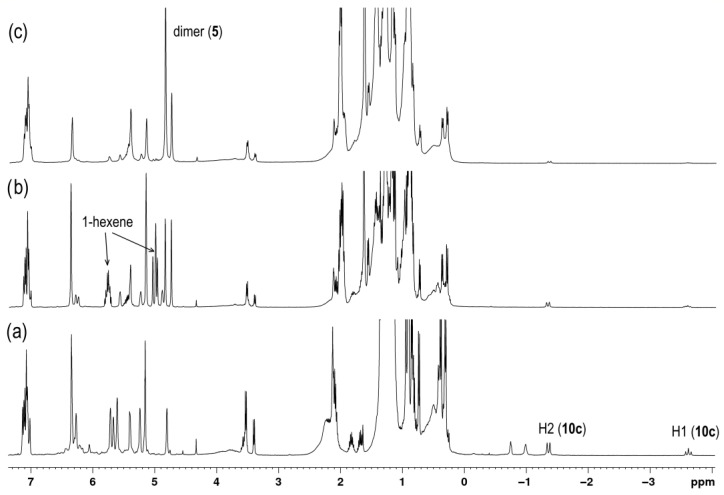
NMR monitoring of the reaction of hydride complexes with 1-hexene in C_6_D_5_CD_3_ (intensity of high-field signals is increased): (**a**) Me_2_CCp_2_ZrCl_2_ (**1c**)−HAlBu^i^_2_–(Ph_3_C)[B(C_6_F_5_)_4_], [Zr]:[Al]:[B] = 1:1:0.1; (**b**) [Zr]:[Al]:[B]:[1-alkene] = 1:1:0.1:1.5, 5 min; (**c**) [Zr]:[Al]:[B]:[1-alkene] = 1:1:0.1:1.5, after keeping the tube at 60 °C for 5 min.

To clarify the reactivity of the complexes towards the alkene, 1-hexene was added to the systems containing various intermediates in different ratios. After the addition of alkene to the reaction media containing an associate of **8c** with MAO, hydrometalation products were formed (Figure S20). The formation of dimers was detected only in systems containing intermediate **10c** when the reaction mixture was heated to 60 °C for several minutes (Figure 3). It is noteworthy that the reaction catalyzed by the cyclopentadienyl complex **1c** at the ratio of reagents [Zr]:[HAlBu^i^_2_]:[(Ph_3_C)(B(C_6_F_5_)_4_)] (MMAO-12) = 1:1:0.1(11), when complexes **9c** and **10c** are present in the catalytic system, was accompanied by a lower conversion of the alkene and the yield of the reaction product **5** (Table 1, entries 9, 10). Apparently, an additional amount of HAlBu^i^_2_ is required both for the preparation of active forms of co-catalysts [34] and for their subsequent reaction with **10c** to give reactive species.

A more pronounced dependency of the reactivity and chemoselectivity of the catalytic system on the composition of the intermediates was observed in the case of the indenyl complex. At a lower content of HAlBu^i^_2_, when both **10f** and cationic species were present in the systems, both dimers and oligomers were found (Table 1, entries 18, 21; Figures S29 and S30). With an increase HAlBu^i^_2_ concentration and, consequently, cationic intermediates, the proportion of oligomers in the reaction products increased (Table 1, entries 19, 22).

Thus, the NMR study of L_2_ZrCl_2_–HAlBu^i^_2_–activator systems demonstrated the formation of various hydride clusters, among which the bis-zirconium hydride intermediate [L_2_ZrH_2_∙L_2_ZrHCl∙ClAlR_2_] may be the precursor of the active sites responsible for dimerization.

## 3. Materials and Methods

### 3.1. General Procedures

All operations for organometallic compounds were performed under argon according to the Schlenk technique. The complexes Cp_2_ZrCl_2_ (**1a**) [47], Me_2_CCpZrCl_2_ (**1c**) [48], Me_2_SiCp_2_ZrCl_2_ (**1d**) [49], (Me_2_C)_2_CpZrCl_2_ (**1e**) [50], Ind_2_ZrCl_2_ (**1f**) [51], *rac*- Me_2_C(Ind)_2_ZrCl_2_ (**1g**) [52], and BIPh(Ind)ZrCl_2_(**1i**) [53] were synthesized from ZrCl_4_. Commercially available compounds, including (C_5_Me_5_)_2_ZrCl_2_ (**1b**) (97%, Acros), *rac*-C_2_H_4_Ind_2_ZrCl_2_ (**1h**) (Strem), *rac*-C_2_H_4_(THInd)_2_ZrCl_2_ (**1j**) (97%, Merck), HAlBu^i^_2_ (99%, Merck), MMAO-12 (7% wt Al in toluene, Merck), (Ph_3_C)[B(C_6_F_5_)_4_] (97%, Abcr), 1-hexene (97%, Acros), were involved into the reactions. The solvents (THF, toluene, benzene) were dried over AlBu^i^_3_ and distilled immediately prior to use.

*CAUTION: pyrophoric nature of aluminum alkyl and hydride compounds require special safety precautions in their handling.*

^1^H and ^13^C NMR spectra were recorded on a Bruker AVANCE-400 spectrometer (400.13 MHz (^1^H), 100.62 MHz (^13^C)) (Bruker, Rheinstetten, Germany). C_7_D_8_ and CDCl_3_ were employed as the solvents and the internal standards. 1D and 2D NMR spectra (COSY HH, HSQC, HMBC, NOESY) were recorded using standard Bruker pulse sequences. 

The analysis of the oligo(1-hexenes) by NMR spectroscopy was carried out according to Refs. [37,38,39,40]. 

The ratio of the light and heavy fractions and the Mw and Mn of oligo(1-hexenes) were determined on a Shimadzu gel permeation chromatograph with a RID-20A detector equipped with a PSS column. Toluene was used as a solvent at a flow rate of 1 mL/min. The measurements were carried out at 25 °C. The standard was polystyrene.

The light products were analyzed using a gas chromatograph-mass spectrometer GCMS-QP2010 Ultra (Shimadzu, Tokyo, Japan) equipped with the GC-2010 Plus chromatograph (Shimadzu, Tokyo, Japan), TD-20 thermal desorber (Shimadzu, Tokyo, Japan), and an ultrafast quadrupole mass-selective detector (Shimadzu, Tokyo, Japan). 

### 3.2. Reaction of L_2_ZrCl_2_ (1a-j) with HAlBu^i^_2_, MMAO-12 or (Ph_3_C)[B(C_6_F_5_)_4_] and 1-Hexene 

A flask with a magnetic stirrer was filled under argon with 10 mg (0.018–0.034 mmol) of L_2_ZrCl_2_, 0.01–0.018 mL (0.055–0.103 mmol) of HAlBu^i^_2_, 0.23–0.44 mL (0.55–1.02 mmol) of MMAO-12, 0.002–0.004 mL (0.018–0.034 mmol) of 1-hexene and 0.5 mL of C_7_D_8_. For organoboron activator (Ph_3_C)[B(C_6_F_5_)_4_], the following amounts were used: 10 mg (0.018–0.034 mmol) of L_2_MCl_2_, 0.0097–0.018 mL (0.0546–0.103 mmol) of HAlBu^i^_2_, 4–63 mg (0.0045–0.0684 mmol) of (Ph_3_C)[B(C_6_F_5_)_4_], 0.002–1.7 mL (0.018–13.7 mmol) of 1-hexene, and 0.5 mL of toluene. The reaction was carried out with stirring at a temperature of 60 °C. After 60 and 180 min, samples (0.1 mL) were syringed into tubes filled with argon, and the samples were decomposed with 10% HCl or DCl at 0 °C. Products were extracted with CH_2_Cl_2_, and the organic layer was dried over Na_2_SO_4_. The yields of products were determined by GC/MS. 

### 3.3. NMR Study of the Reaction of Me_2_CCp_2_ZrCl_2_ or Ind_2_ZrCl_2_ with HalBu^i^_2_ and Activator (MMAO-12, (Ph_3_C)[B(C_6_F_5_)_4_])

The NMR tube was charged with 0.029–0.045 mmol of Me_2_CCp_2_ZrCl_2_ or Ind_2_ZrCl_2_ and C_7_D_8_ in an argon-filled glovebox. The tube was cooled to 0 °C, and 0.03–0.25 mmol (3.8–36 mg) of HAlBu^i^_2_ was added dropwise. The mixture was stirred, and the formation of complexes **8**–**10** was monitored by NMR. Then, 0.17–0.34 mmol (65.4–0.131 mg) of MMAO-12 or 0.005–0.022 mmol (4.7–20 mg) of (Ph_3_C)[B(C_6_F_5_)_4_] were added.

## 4. Conclusions

To reveal the regularity of metallocene-AOC-activator catalytic systems’ action, we studied the activity and chemo- and stereoselectivity of *η^5^*-zirconium complexes in the presence of HAlBu^i^_2_, MMAO-12 or [Ph_3_C][B(C_6_F_5_)_4_] in 1-hexene oligomerization. Depending on the composition of the catalytic system, the formation of 1-hexene vinylidene dimer and oligomers, 1,2-insertion products with different double bond positions, were observed.

The dimerization pathway is implemented, as a rule, in the presence of Zr complexes with sterically non-hindered cyclopentadienyl ligands (L = Cp, *ansa*-Me_2_CCp_2_, *ansa*-(Me_2_C)_2_Cp_2_, *ansa*-Me_2_SiCp_2_). The use of zirconocenes with bulky cyclopentadienyl (L = C_5_Me_5_, *rac*-H_4_C_2_[THInd]_2_) or electron-withdrawing indenyl (L = Ind, Me_2_CInd_2_, H_4_C_2_Ind_2_, BIPh(Ind)_2_) ligands resulted in the formation of oligomers.

Indenyl complexes (L = Ind, Me_2_CInd_2_, H_4_C_2_[Ind]_2_) showed the highest values of isotacticity at 67, 93, 71% mmmm, respectively. Moreover, stereoselectivity was notably dependent on the type of activator, suggesting a significant influence of the co-catalyst on the stereoregulation process in the course of alkene coordination by the catalytically active species.

Considerable differences in catalyst action are indicative of the realization of dimerization and oligomerization processes through different active centers, including hydride intermediates. In the example of the reaction of *ansa*-Me_2_CCp_2_ZrCl_2_ and Ind_2_ZrCl_2_ with HAlBu^i^_2_ and activators, the composition of hydride intermediates was studied by NMR spectroscopy. As a result, the bis-zirconium complex of type [L_2_ZrH_2_∙L_2_ZrHCl∙ClAlR_2_], which is the precursor of catalytically active centers of the alkene dimerization, was found.

## Figures and Tables

**Table 1:** Catalytic activity and chemoselectivity of L_2_ZrCl_2_–HAlBu^i^_2_–activator systems in the reaction with 1-hexene in toluene, 60 °C.

Entry	Catalytic Systems		Mole Ratio [Zr]: [HAlBu^i^_2_]: [Activator]: [1-Alkene]	Time, Min	Alkene Conver-sion, %	Light Fraction Yield, wt.%	Light Fraction Product Composition, % (GC-MS)						Heavy Fraction Yield, wt.% (GPC)	M_W_, Da	M_N_, Da	M_W_/ M_N_	Tacticity, mmmm%	Oligomer Structural Type
	Complex	Activator					5	6										
								*n* = 1	*n* = 2	*n* = 3	*n* = 4	*n* = 5						
1	Cp_2_ZrCl_2_ (**1a**)	(Ph_3_C)[B(C_6_F_5_)_4_]	1:3:0.3:400	60	95	98	61	21	10	6	2	-	2	6211	6129	1.01	atactic	**6b**–**d**
2			1:3:1:400		>99	99	30	28	20	10	8	4	-					
3			1:3:2:400		>99 ^a^								33	5277	5208	1.01		
4			4:16:1:400		>99	88	61	5	12	8	8	5	2	5571	5459	1.02		
													10	1578	1211	1.30		
5		MMAO-12 [31]	1:3:30:400	5	98		97						-					
6	(C_5_Me_5_)_2_ZrCl_2_ (**1b**)	(Ph_3_C)[B(C_6_F_5_)_4_]	4:16:1:400	180	99	44	0	27	18	30	24		55	1109	933	1.19	atactic	**6a**
7		MMAO-12	1:3:30:400	60	99	3	28	23	17	16	16		71	5605	5906	1.05	atactic	**4, 6a ** ^d^
													26	861	798	1.08		
8	Me_2_CCp_2_ZrCl_2_ (**1c**)	(Ph_3_C)[B(C_6_F_5_)_4_]	4:16:1:400	60	>99		95	4					-					
9			1:1:0.1:400	180	91		86	5										
10		MMAO-12	1:3:30:400	60	>99		94	6					-					
11			1:1:11:400	180	77		75	2										
12	Me_2_SiCp_2_ZrCl_2_ (**1d**)	(Ph_3_C)[B(C_6_F_5_)_4_]	4:16:1:400	180	>99	5	17	28	15	21	18		89	6130	6041	1.02	atactic	**6a, 6d**
													6	2769	2672	1.04		
13		MMAO-12	1:3:30:400	60	>99	99	80	16	4				-					
14	(Me_2_C)_2_Cp_2_ZrCl_2_ (**1e**)	(Ph_3_C)[B(C_6_F_5_)_4_]	4:16:1:400	60	>99	99	98	1					-					
15		MMAO-12	1:3:30:400	60	85		78	7					-					
16	Ind_2_ZrCl_2_ (**1f**)	(Ph_3_C)[B(C_6_F_5_)_4_]	1:3:0.3:400	180	>99	96	7	23	32	21	17		3	6307	6200	1.02	atactic	**6a**–**d**
17			4:16:1:400	60	>99	63	0	22	20	26	31		36	988	869	1.14	atactic	**6a**
18 ^e^			1:3:0.2:5	10	74 ^f^		12	16	16									
19 ^e^			1:8:0.2:80	10	>99 ^g^		13	16	16	10	9	8						
20		MMAO-12	1:3:30:400	60	>99	27	6	15	17	19	43		72	2182	1599	1.36	isotactic (67%)	**4, 6a,d**
21 ^e^			1:3:7:3	10	>99 ^h^		46	8										
22 ^e^			1:8:10:80	10	>99 ^i^		27	22	17	8	4							
23	*rac*-Me_2_CInd_2_ZrCl_2_ (**1g**)	(Ph_3_C)[B(C_6_F_5_)_4_]	1:3:0.3:400	60	97	70	22	21	20	19	18		27	6359	6291	1.01	isotactic (93%)	**6d**
24			4:16:1:400	60	>99	72	79	10	5	4	2		24	5585	5144	1.08	isotactic (76%)	**6a**–**d**
													3	903	871	1.04		
25		MMAO-12	1:3:30:400	60	>99	55	4	33	33	18	12		44	1081	927	1.17	atactic	**4, 6a**–**d** ^d^
26	*rac*-H_4_C_2_Ind_2_ZrCl_2_ (**1h**)	(Ph_3_C)[B(C_6_F_5_)_4_]	4:16:1:400	60	>99								99	6396	6285	1.02	isotactic (71%)	**6a**–**d**
27		MMAO-12	1:3:30:400	60	>99	5	7	22	22	24	25		94	6347	6287	1.01	isotactic (61%)	**4, 6a,d**
28	BIPh(Ind)_2_ZrCl_2_ (**1i**)	(Ph_3_C)[B(C_6_F_5_)_4_]	1:3:0.3:400	180	47 ^b^	25	13	49	23	15			22	6037	6125	1.01		
29			4:16:1:400	60	>99 ^b^	59	7	21	26	14	32		40	1096	1003	1.09	isotactic (33%)	**6a**
30		MMAO-12	1:3:30:400	60	>99 ^b^	9	3	20	25	23	29		20	5742	5657	1.02	isotactic (45%)	**4, 6a ** ^d^
													70	2159	1532	1.4		
31	*rac*-H_4_C_2_[THInd]_2_ZrCl_2_ (**1j**)	(Ph_3_C)[B(C_6_F_5_)_4_]	1:3:0.3:400	180	89 ^c^	69	13	18	20	15	14	12	10	6131	6048	1.02		
													10	960	909	1.06		
32			4:16:1:400	180	>99	11	29	19	18	17	17		88	5530	5033	1.10	isotactic (38%)	**6a**–**d**
33		MMAO-12	1:3:30:400	180	95	8	11	24	24	23	18		38	6159	6103	1.01	isotactic (58%)	**6a**–**d**
													19	3145	3114	1.01		
													30	2074	1971	1.06		

^a^ Monoalkyl-substituted toluene (77%), dialkyl-substituted toluene (19%), and trialkyl-substituted toluene (4%) were detected; ^b^ tetramers, that is, “dimer of dimers” (15%), were formed in the presence of both activators; ^c^ the yield of oligomers with *n* = 6 were up to 7%; ^d^ Me starting group; ^e^ reaction was carried out in an NMR tube at 20 °C; ^f^ hydrometalation product (8%), oligomer **4** (m = 1: 15%, m = 2: 7%, R = H); ^g^ hydrometalation product (4%), oligomer **4** (m = 1: 18%, R = H); ^h^ hydrometalation product (22%), oligomer **4** (m = 1: 24%, R = H); ^i^ hydrometalation product (4%), oligomer **4** (m = 1: 18%, R = H).

**Table 2:** ^1^H and ^13^C NMR (δ, ppm, 400.13 MHz (^1^H), 100.62 MHz (^13^C)) of hydride complexes.

Complex	Activator	T, K	δ_H_ Cp	δ_C_ Cp	δ_H_ H^1^	δ_H_ H^2^
**8c**		250 K	6.11 (s, 4H) 5.19 (s, 4H) 1.03 (s, 12H)	110.9 110.3 100.8 36.8 22.3	−1.44 (br.s, 2H)	1.29–1.43 (br.s, 1H)
**9c**			6.28 (s, 2H) 6.23 (s, 2H) 5.66 (s, 2H) 5.37 (s, 2H) 1.15 (NOESY) 1.12 (NOESY)		−1.10 (br.s, 1H)	−0.81 (br.s, 1H)
**10c**		298 K			−3.58 (t, 17.9 Hz, 1H)	−1.34 (d, 17.9 Hz, 2H)
**8c∙MAO**	MMAO−12	298 K	6.14 (NOESY) 4.93–5.69 (br.m)		−1.62− −1.22 (br.s)	1.25–1.48 (COSY HH) −0.38 − −0.02 (MAO) 3.26–4.11 (H-MAO)
**10c∙MAO**	MMAO−12	298 K	6.12 (NOESY) 5.67 (NOESY) 5.42 (NOESY) 4.85 (NOESY) 0.98 (NOESY)	119.6 108.7 101.2 98.2 21.1	−3.57 (t, 17.6 Hz, 2/3H) −3.71 (t, 18.0 Hz, 1/3H)	−1.32 (d, 17.6 Hz, 2H) −0.56 − 0.15 (MAO)
**10c ^a^**	[Ph_3_C][B(C_6_F_5_)_4_]	298 K			−3.59 (t, 18.3 Hz, 2H)	−1.34 (d, 18.3 Hz, 2H)
**8f** [43]		220 K	7.37 (m, 4H) 6.87 (m, 4H) 6.32 (m, 2H) 5.19 (m, 4H)		−1.11 (d, 5.6 Hz, 2H)	0.07 (t, 7.6 Hz, 1H)
**8f**		292 K	7.43 (m, 4H) 6.89 (m, 4H) 6.38 (m, 2H) 5.36 (m, 4H)		−0.92 (br.s, 2H)	0.07 (NOESY)
**10f∙MAO**	MMAO−12	298 K	5.92 (m, 4H) 5.08 (m, 4H) 4.62 (m, 4H)		−6.11 (t, 18.5 Hz, 1H)	0.05 (COSY HH)
**10f ^a^**	[Ph_3_C][B(C_6_F_5_)_4_]	298 K	7.49 (d, 8.3 Hz, 4H) 7.19 (d, 8.3 Hz, 4H) 6.99–7.16 (m, 8H) 5.82 (br.t, 3.0 Hz, 4H) 5.02 (m, 4H) 4.57 (m, 4H)	127.9 126.6 119.0 117.3 99.1 96.2	−6.00 (t, 18.1 Hz, 1H)	−0.04 (d, 18.1 Hz, 2H)

^a^ The complex was observed after the addition of [Ph_3_C][B(C_6_F_5_)_4_].

## Data Availability

Not applicable.

## Supplementary Materials

The following supporting information can be downloaded at: https://www.mdpi.com/article/10.3390/molecules28062420/s1, Figures S1–S30: NMR spectra of reaction products and intermediates.
