# Supplementary material for: Catalytic Properties of Zirconocene-Based Systems in 1-Hexene Oligomerization and Structure of Metal Hydride Reaction Centers
Source: Molecules. 2023 Mar 7;28(6):2420. doi: 10.3390/molecules28062420 (PMC10058051; doi:10.3390/molecules28062420)

Catalytic Properties of Zirconocene-Based Systems in 1-Hexene Oligomerization and Structure of Metal Hydride Reaction Centers

Lyudmila V. Parfenova [1,*], Pavel V. Kovyazin [1], Almira Kh. Bikmeeva [1], Eldar R. Palatov [1], Pavel V. Ivchenko [2,3], Ilya E. Nifant'ev [2,3] and Leonard M. Khalilov [1]

[1] Institute of Petrochemistry and Catalysis, Ufa Federal Research Center, Russian Academy of Sciences, Prosp. Oktyabrya, 141, 450075 Ufa, Russia

[2] Department of Chemistry, Lomonosov Moscow State University, 1-3 Leninskiye Gory, 119991 Moscow, Russia

[3] A.V. Topchiev Institute of Petrochemical Synthesis, Russian Academy of Sciences, Leninsky Prosp. 29, 119991 Moscow, Russia

***** Correspondence: luda_parfenova@ipc-ras.ru

Supporting Information

Figure S1. ^{1}H and ^{13}C NMR of 1-hexene oligomers obtained in the system *rac*-$Me_2CInd_2ZrCl_2$ (**1g**) − $HAlBu^i_2$ − $(Ph_3C)[B(C_6F_5)_4]$ 3

Figure S2. ^{1}H and ^{13}C NMR of 1-hexene oligomers obtained in the system *rac*-$H_4C_2[Ind]_2ZrCl_2$ (**1h**)- $HAlBu^i_2$-MMAO-12 4

Figure S3. Comparison of ^{13}C NMR of 1-hexene oligomers obtained in the system Ind_2ZrCl_2 (**1f**)- $HAlBu^i_2$ activated by MMAO-12 and $(Ph_3C)[B(C_6F_5)_4]$. 5

Figure S4. Comparison of ^{13}C NMR of 1-hexene oligomers obtained in the system *rac*-$Me_2CInd_2ZrCl_2$ (**1g**)- $HAlBu^i_2$ activated by MMAO-12 and $(Ph_3C)[B(C_6F_5)_4]$. 5

Figure S5. ^{1}H NMR of system $Me_2CCp_2ZrCl_2$ (**1c**) − $HAlBu^i_2$ (1:3) in C_7D_8 (250 K). 6

Figure S6. COSY HH of system $Me_2CCp_2ZrCl_2$ (**1c**) − $HAlBu^i_2$ (1:3) in C_7D_8 (250 K). 6

Figure S7. NOESY of system $Me_2CCp_2ZrCl_2$ (**1c**) − $HAlBu^i_2$ (1:3) in C_7D_8 (250 K). 7

Figure S8. NOESY of system $Me_2CCp_2ZrCl_2$ (**1c**) − $HAlBu^i_3$ (1:8) in C_7D_8 (298 K). 7

Figure S9. ^{1}H NMR of system $Me_2CCp_2ZrCl_2$ (**1c**) − $HAlBu^i_2$ (1:1.7) in C_7D_8 (298 K). 8

Figure S10. COSY HH of system $Me_2CCp_2ZrCl_2$ (**1c**) − $HAlBu^i_2$ (1:1.7) in C_7D_8 (298 K). 8

Figure S11. ^{1}H NMR of system MMAO-12 − $HAlBu^i_2$ (1:3) in C_7D_8 (298 K). 9

Figure S12. NOESY of system MMAO-12 − $HAlBu^i_2$ (1:3) in C_7D_8 (298 K). 9

Figure S13. ^{1}H NMR of system $Me_2CCp_2ZrCl_2$ (**1c**) − $HAlBu^i_2$ − MMAO-12 (1:8:11) in C_7D_8 (298 K). 10

Figure S14. NOESY of system $Me_2CCp_2ZrCl_2$ (**1c**) − $HAlBu^i_2$ − MMAO-12 (1:8:11) in C_7D_8 (298 K). 10

Figure S15. ^{1}H NMR of system $Me_2CCp_2ZrCl_2$ (**1c**) − $HAlBu^i_2$ – MMAO-12 (1:1.7:7) in C_7D_8 (298 K). 11

Figure S16. COSY HH of system $Me_2CCp_2ZrCl_2$ (**1c**) − $HAlBu^i_2$ – MMAO-12 (1:1.7:7) in C_7D_8 (298 K). 11

Figure S17. NOESY of system $Me_2CCp_2ZrCl_2$ (**1c**) − $HAlBu^i_2$ – MMAO-12 (1:1.7:7) in C_7D_8 (298 K). 12

Figure S18. ^{1}H NMR of system $Me_2CCp_2ZrCl_2$ (**1c**) − $HAlBu^i_2$ – $(Ph_3C)[B(C_6F_5)_4]$ (1:1:0.1) in C_7D_8 (298 K). 13

Figure S19. COSY HH of system $Me_2CCp_2ZrCl_2$ (**1c**) − $HAlBu^i_2$ – $(Ph_3C)[B(C_6F_5)_4]$ (1:1:0.1) in C_7D_8 (298 K). 13

Figure S20. NMR monitoring of system $Me_2CCp_2ZrCl_2$ (**1c**) − $HAlBu^i_2$ − MMAO-12 – 1-hexene (1:8:11:2) in C_7D_8. 14

Figure S21. ^{1}H NMR of system Ind_2ZrCl_2 (**1f**) − $HAlBu^i_2$ (1:8) in C_7D_8: a) 220 K [43], b) 298 K. 15

Figure S22. NOESY of system Ind_2ZrCl_2 (**1f**) − $HAlBu^i_2$ (1:3) in C_7D_8 (298 K). 15

Figure S23. ^{1}H NMR of system Ind_2ZrCl_2 (**1f**) − $HAlBu^i_2$ – $(Ph_3C)[B(C_6F_5)_4]$ (1:3:0.2) in C_7D_8 (298 K). 16

Figure S24. COSY HH of system Ind_2ZrCl_2 (**1f**) − $HAlBu^i_2$ – $(Ph_3C)[B(C_6F_5)_4]$ (1:3:0.2) in C_7D_8 (298 K). 16

Figure S25. ^{1}H NMR of system Ind_2ZrCl_2 (**1f**) − $HAlBu^i_2$ – $(Ph_3C)[B(C_6F_5)_4]$ (1:8:0.2) in C_7D_8 (298 K). 17

Figure S26. ^{1}H NMR of system Ind_2ZrCl_2 (**1f**) − $HAlBu^i_2$ – MMAO-12 (1:3:7) in C_7D_8 (298 K). 17

Figure S27. COSY HH of system Ind_2ZrCl_2 (**1f**) − $HAlBu^i_2$ – MMAO-12 (1:3:7) in C_7D_8 (298 K). 18

Figure S28. ^{1}H NMR of system Ind_2ZrCl_2 (**1f**) − $HAlBu^i_2$ – MMAO-12 (1:7:15) in C_7D_8 (298 K). 18

Figure S29. ^{13}C NMR of products obtained in the system Ind_2ZrCl_2 (**1f**) − $HAlBu^i_2$ − $(Ph_3C)[B(C_6F_5)_4]$ – 1-hexene in C_7D_8 at 298 K: a) [Zr]:[$HAlBu^i_2$]:[B]:[1-hexene]=1:3:0.2:5, 10 min; b) [Zr]:[$HAlBu^i_2$]:[B]:[1-hexene]=1:8:0.2:80, 10 min. 19

Figure S30. ^{13}C NMR of products obtained in the system Ind_2ZrCl_2 (**1f**) − $HAlBu^i_2$ – MMAO-12 – 1-hexene in C_7D_8 at 298 K: a) [Zr]:[$HAlBu^i_2$]:[Al_{MAO}]:[1-hexene]=1:3:7:3, 10 min; b) [Zr]:[$HAlBu^i_2$]:[Al_{MAO}]:[1-hexene]=1:8:10:80, 10 min. 20

Figure S1. ^{1}H and ^{13}C NMR of 1-hexene oligomers obtained in the system *rac*-$Me_2CInd_2ZrCl_2$ (**1g**) − $HAlBu^i_2$ − $(Ph_3C)[B(C_6F_5)_4]$

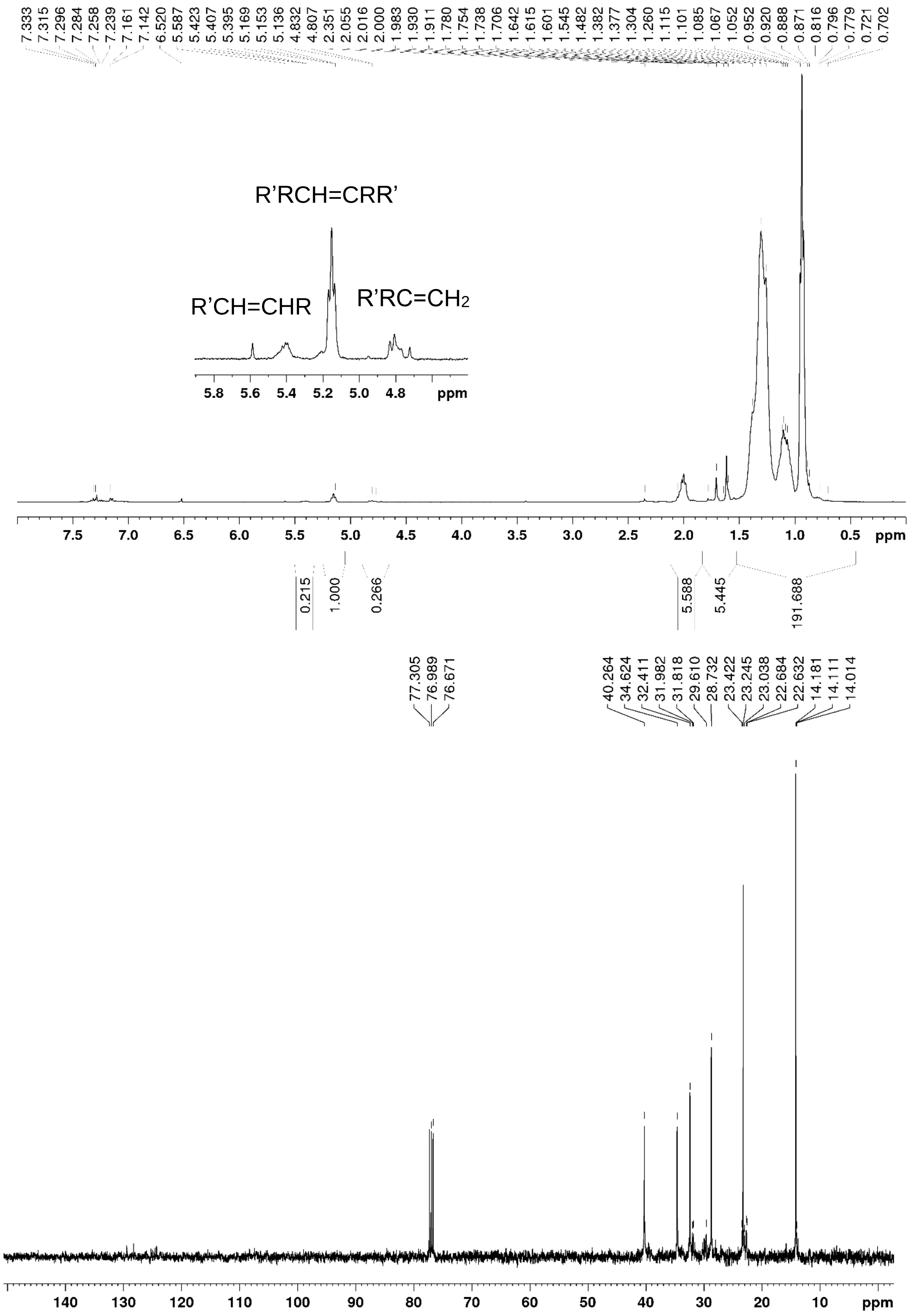

Figure S2. ^{1}H and ^{13}C NMR of 1-hexene oligomers obtained in the system *rac*-$H_4C_2[Ind]_2ZrCl_2$ (**1h**)- $HAlBu^i_2$-MMAO-12

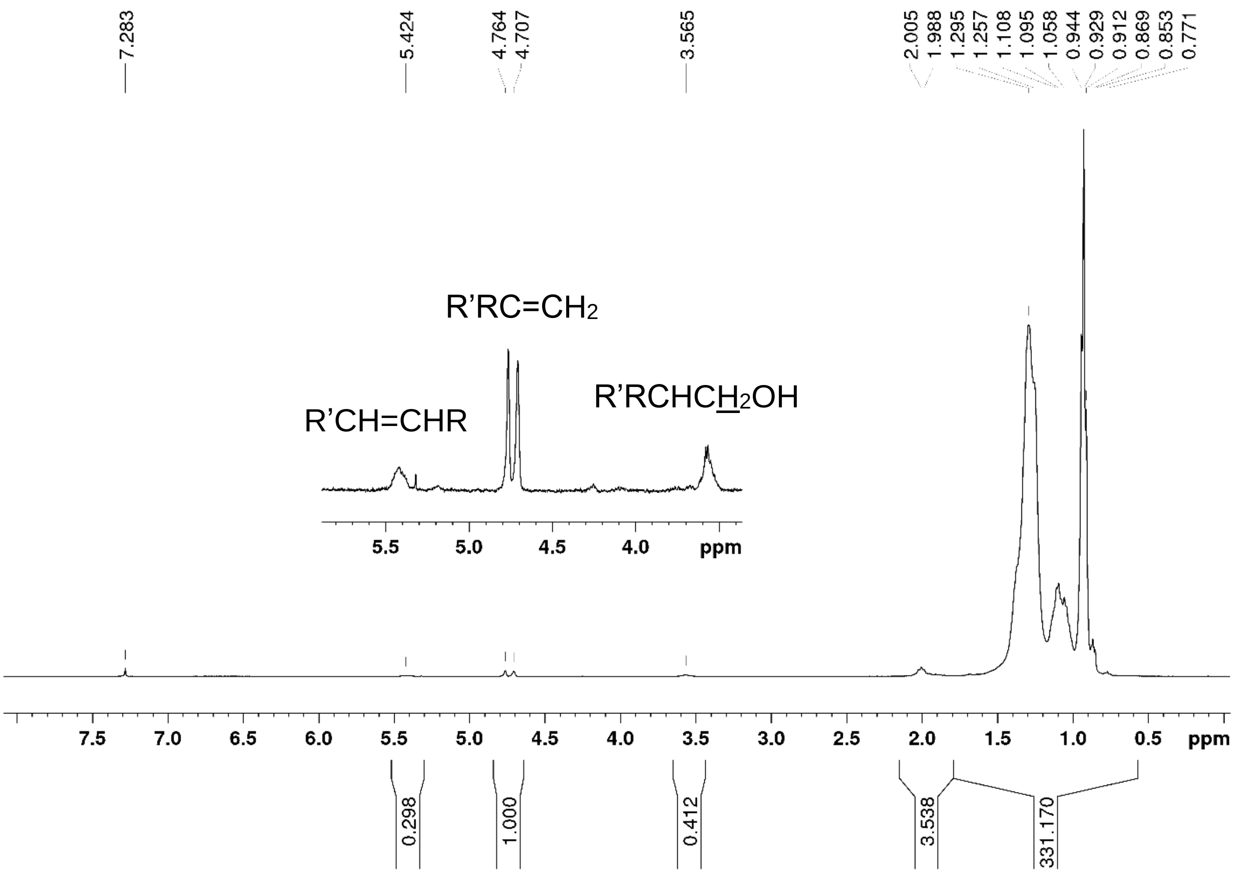

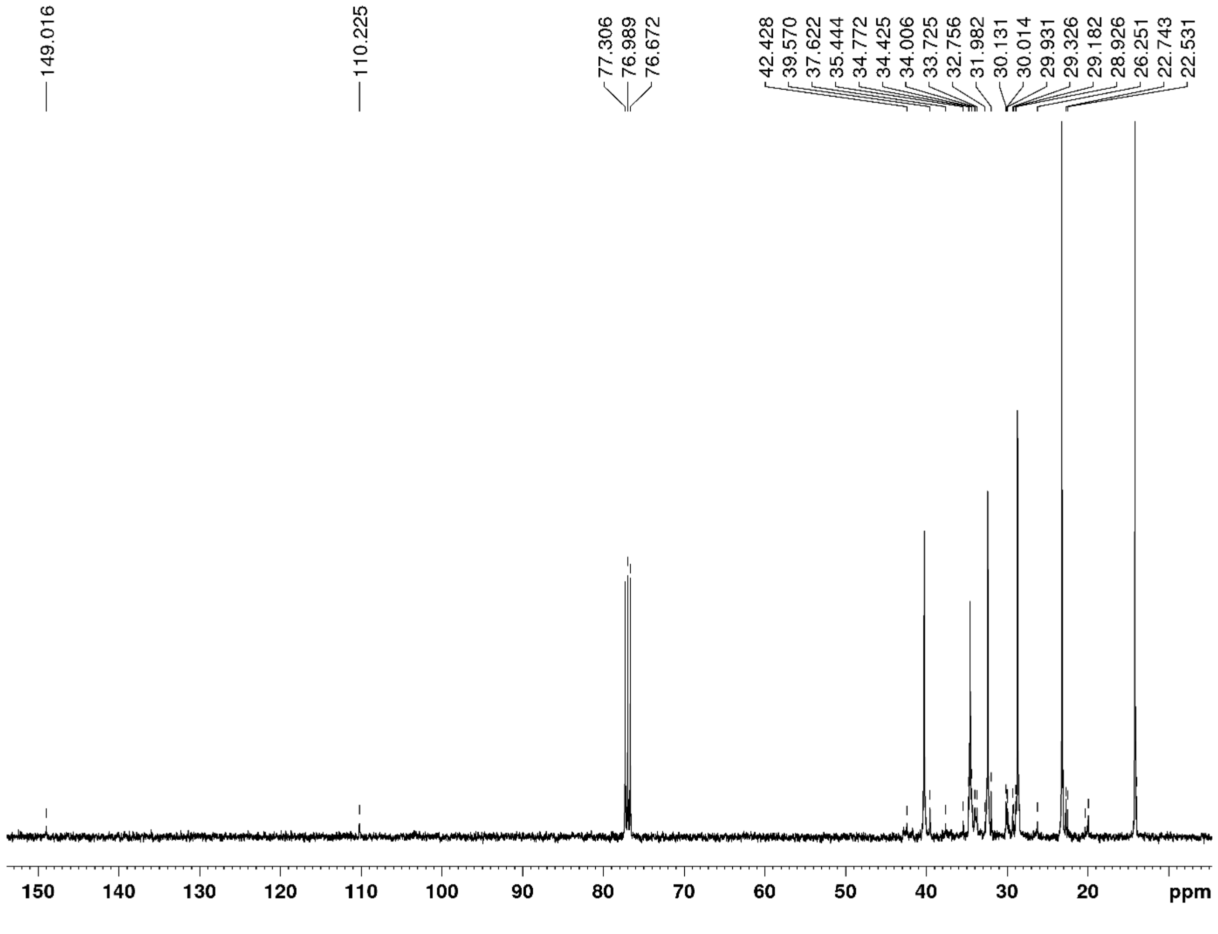

Figure S3. Comparison of ^{13}C NMR of 1-hexene oligomers obtained in the system Ind_2ZrCl_2 (**1f**)- $HAlBu^i_2$ activated by MMAO-12 and $(Ph_3C)[B(C_6F_5)_4]$.

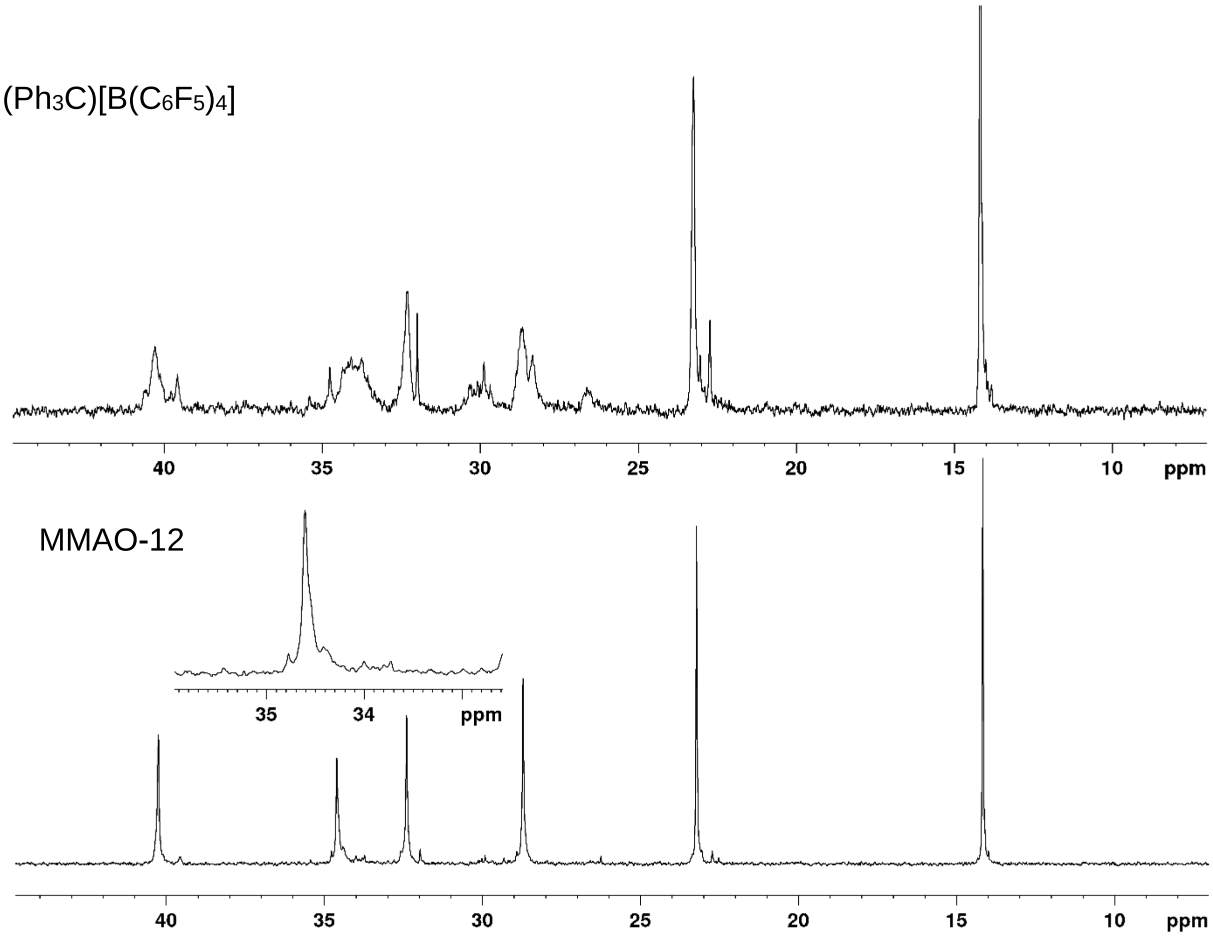

Figure S4. Comparison of ^{13}C NMR of 1-hexene oligomers obtained in the system *rac*-$Me_2CInd_2ZrCl_2$ (**1g**)- $HAlBu^i_2$ activated by MMAO-12 and $(Ph_3C)[B(C_6F_5)_4]$.

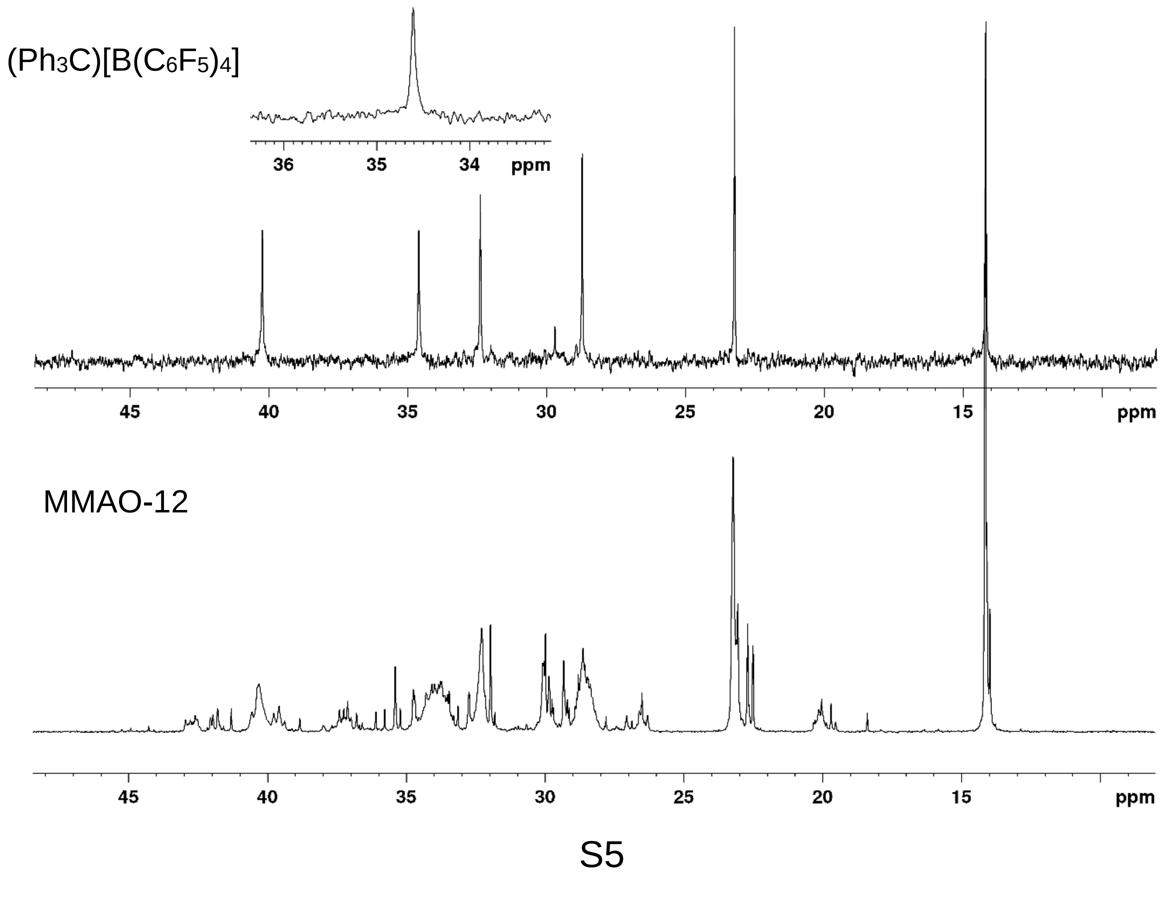

Figure S5. ^{1}H NMR of system $Me_2CCp_2ZrCl_2$ (**1c**) − $HAlBu^i_2$ (1:3) in C_7D_8 (250 K).

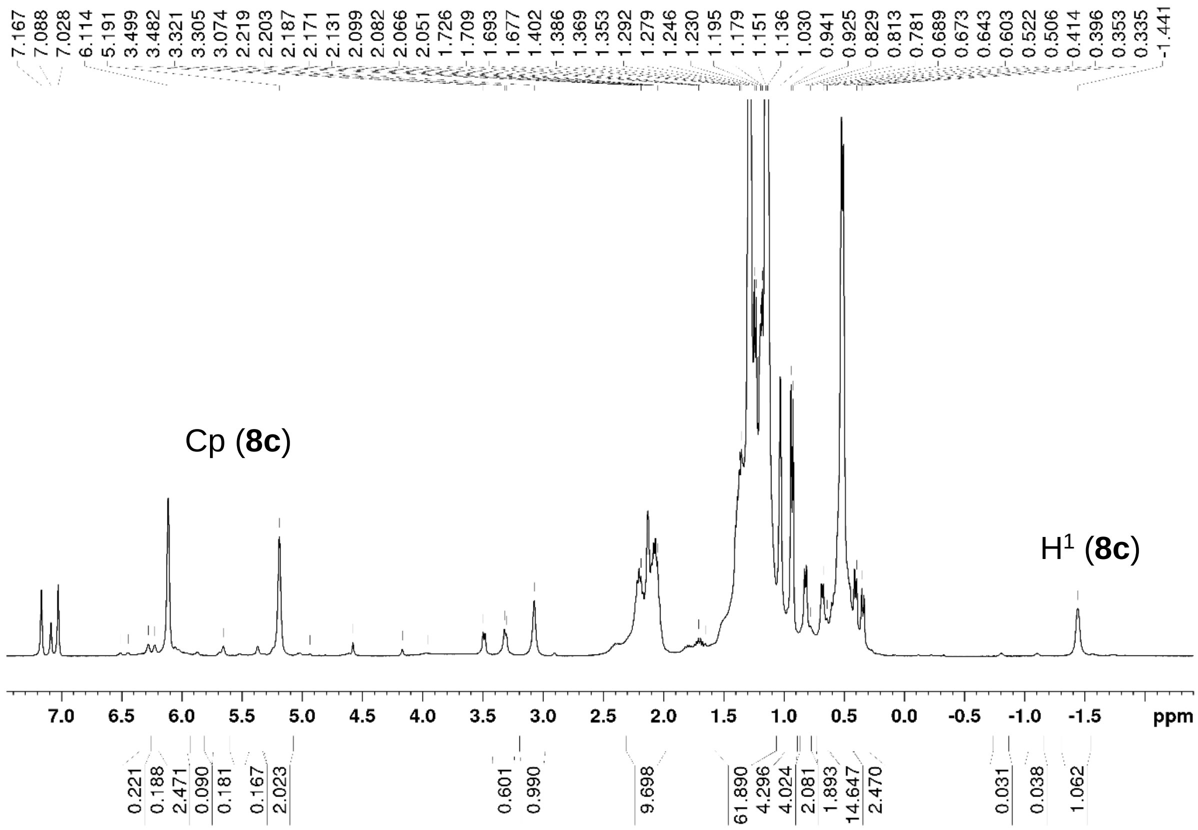

Figure S6. COSY HH of system $Me_2CCp_2ZrCl_2$ (**1c**) − $HAlBu^i_2$ (1:3) in C_7D_8 (250 K).

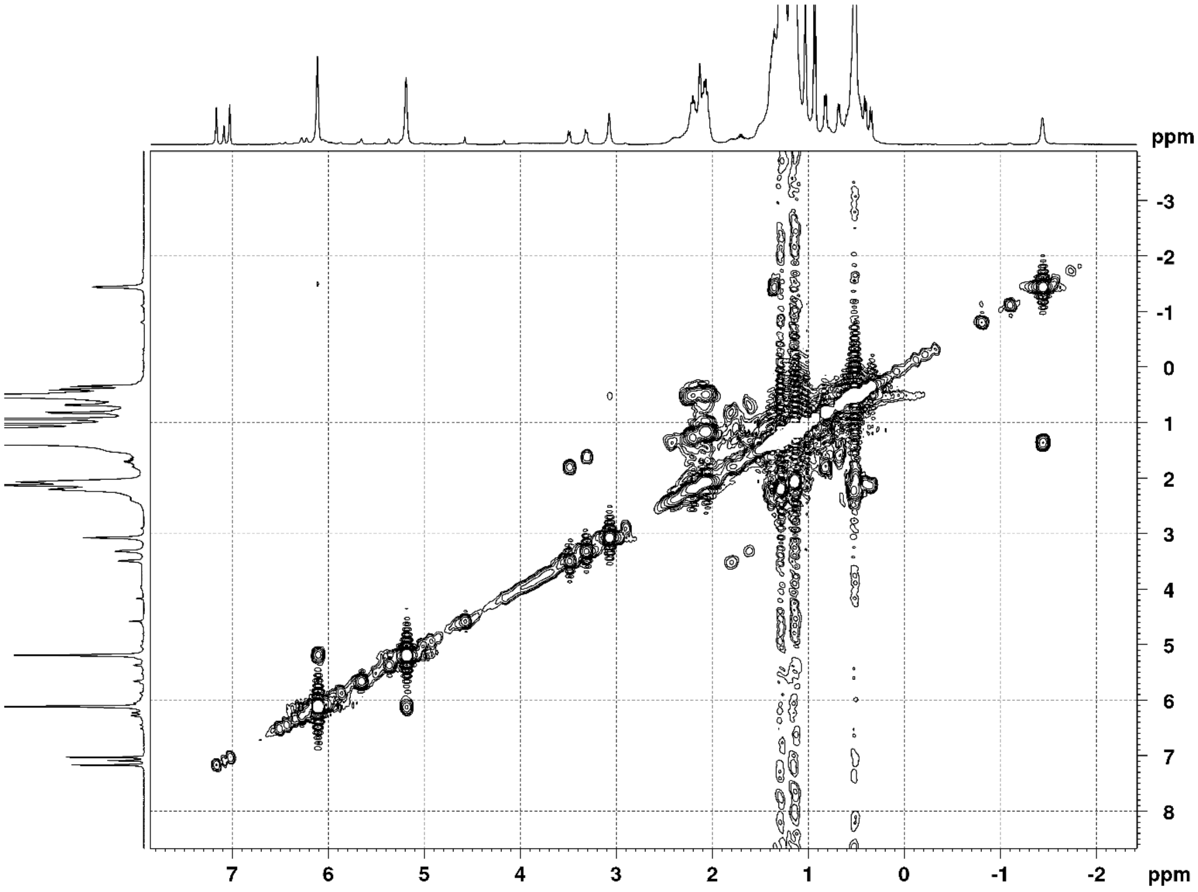

Figure S7. NOESY of system $Me_2CCp_2ZrCl_2$ (**1c**) − $HAlBu^i{}_2$ (1:3) in C_7D_8 (250 K).

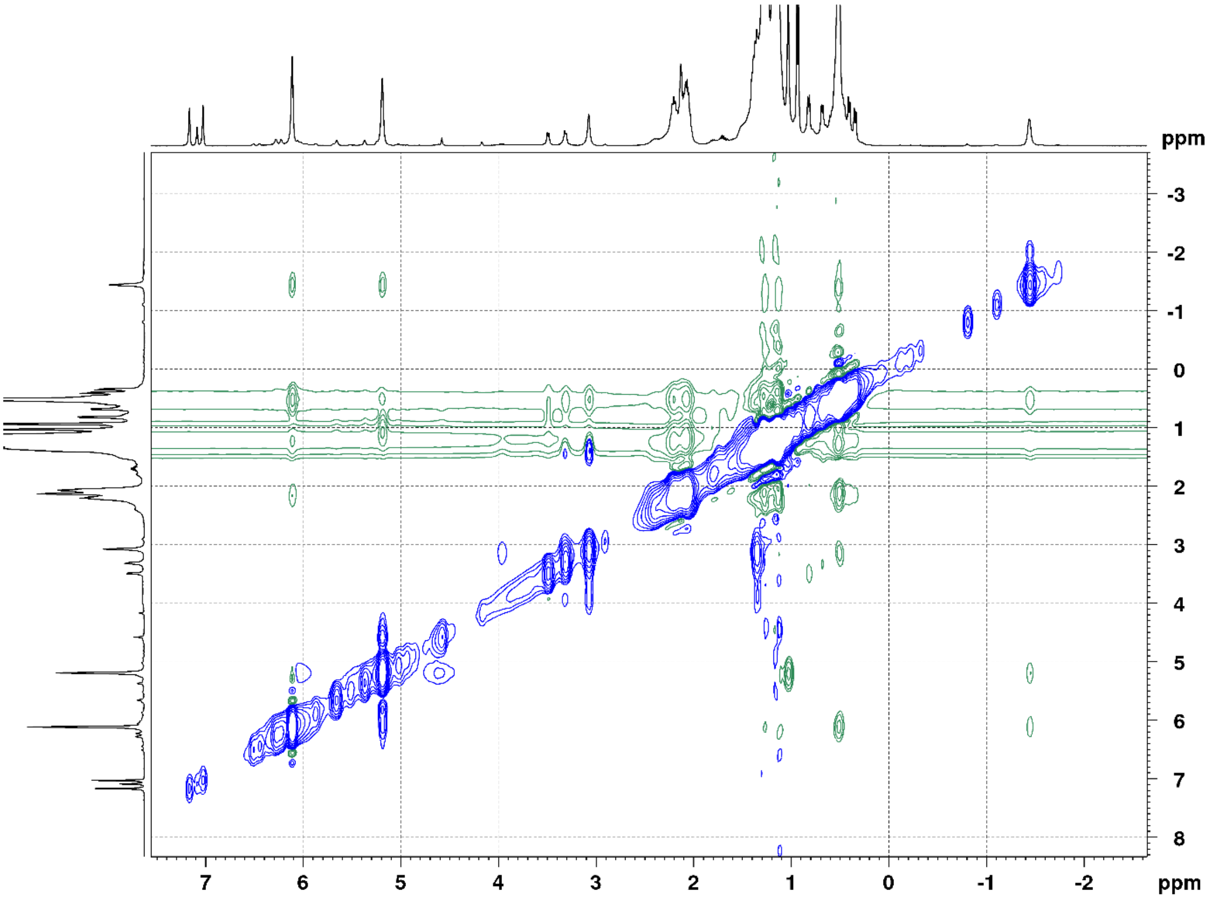

Figure S8. NOESY of system $Me_2CCp_2ZrCl_2$ (**1c**) − $HAlBu^i{}_3$ (1:8) in C_7D_8 (298 K).

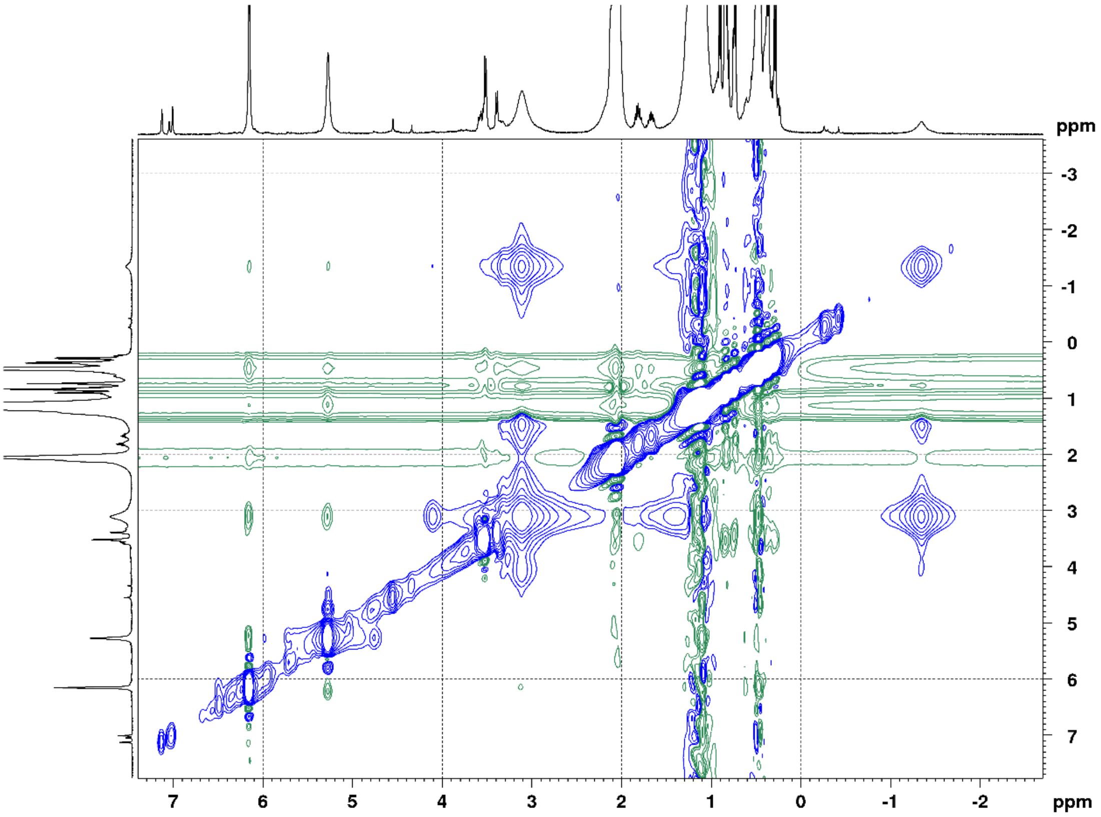

Figure S9. ^{1}H NMR of system $Me_2CCp_2ZrCl_2$ (**1c**) − $HAlBu^i_2$ (1:1.7) in C_7D_8 (298 K).

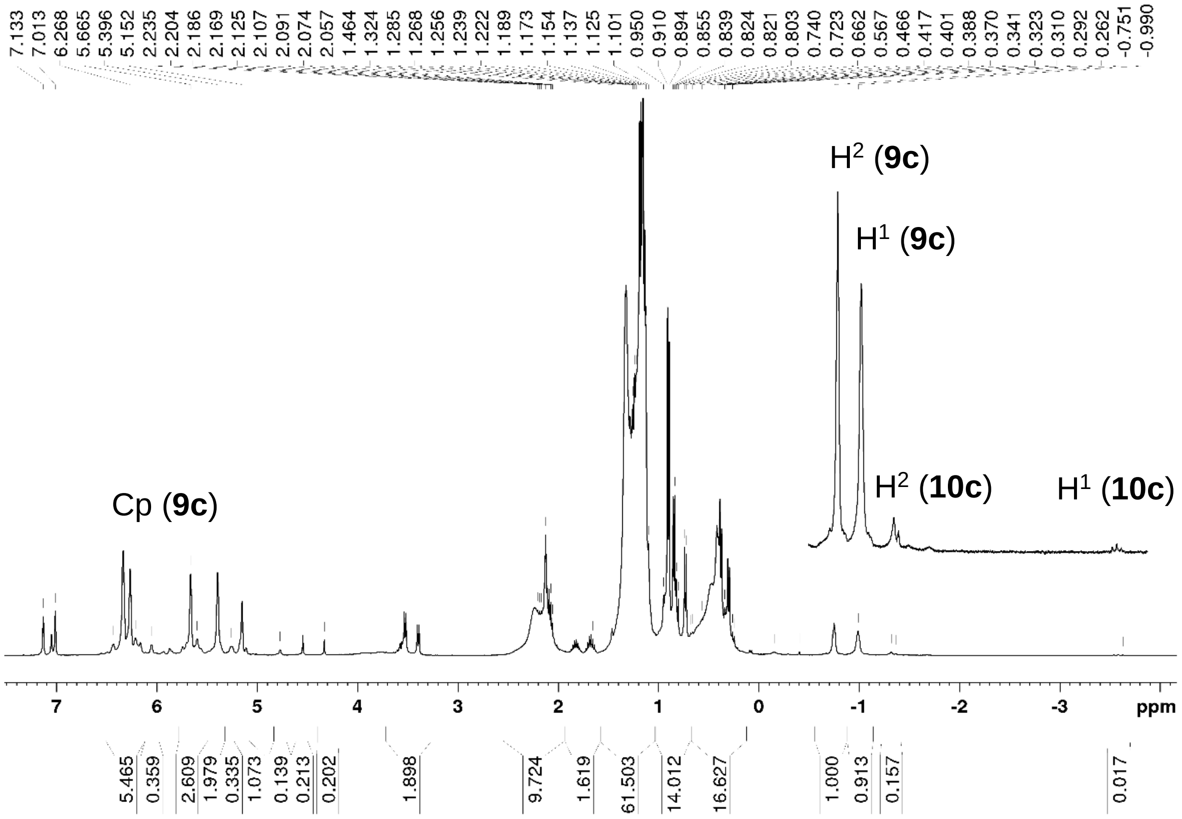

Figure S10. COSY HH of system $Me_2CCp_2ZrCl_2$ (**1c**) − $HAlBu^i_2$ (1:1.7) in C_7D_8 (298 K).

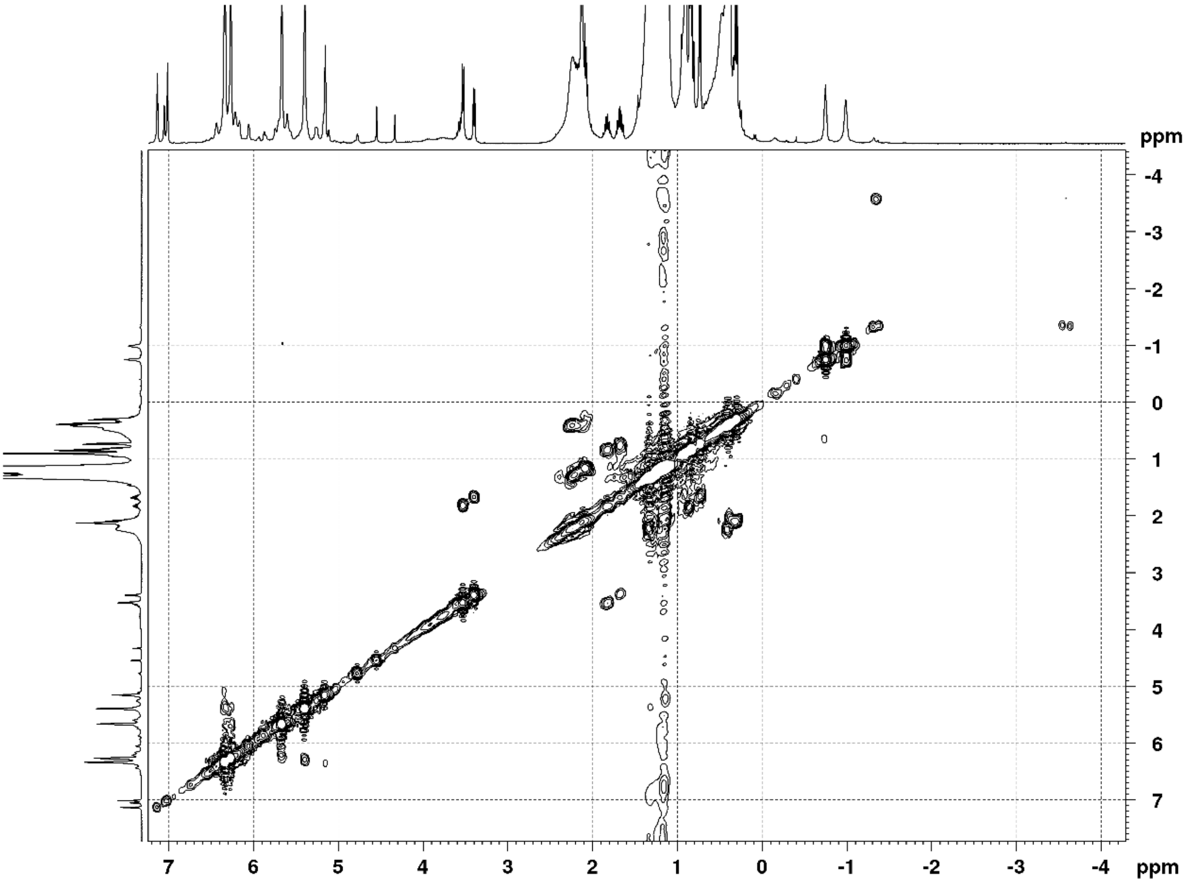

Figure S11. ^{1}H NMR of system MMAO-12 − $HAlBu^{i}_{2}$ (1:3) in C_7D_8 (298 K).

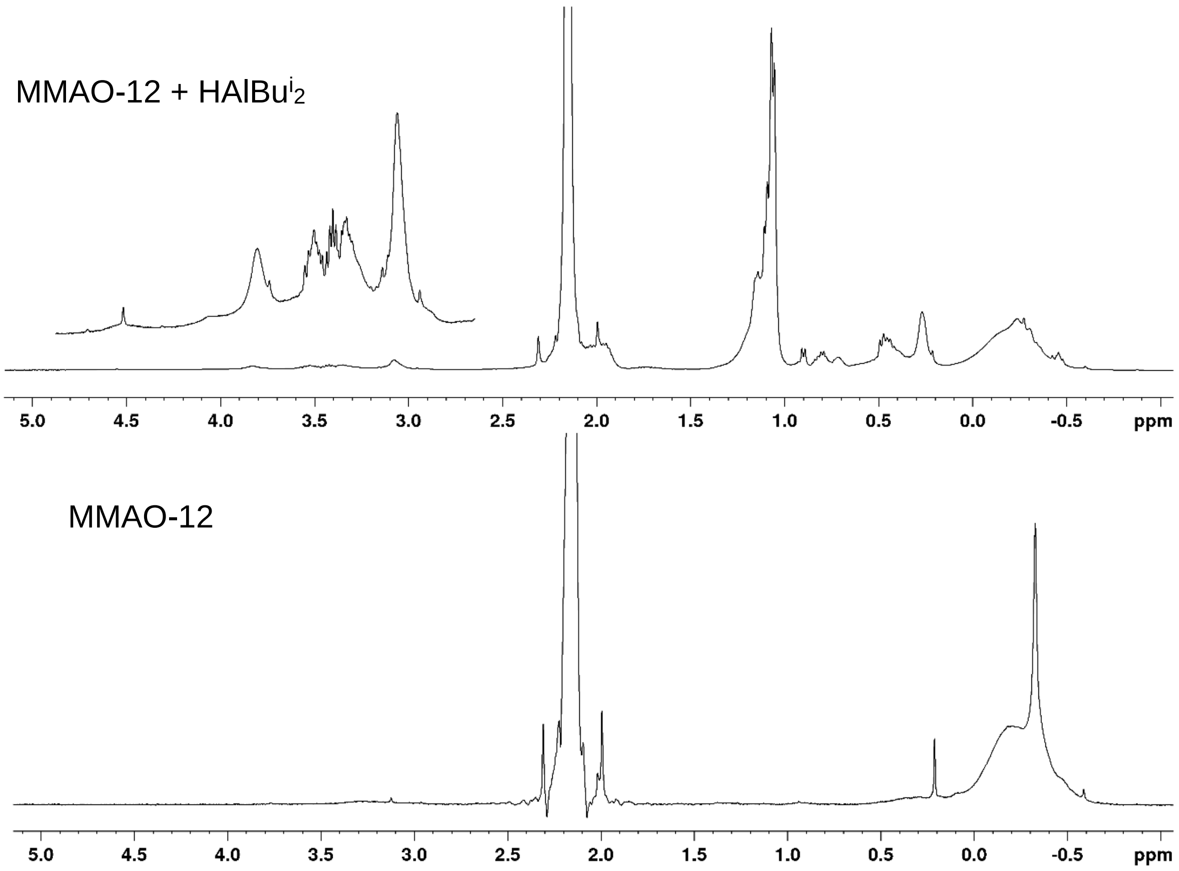

Figure S12. NOESY of system MMAO-12 − $HAlBu^{i}_{2}$ (1:3) in C_7D_8 (298 K).

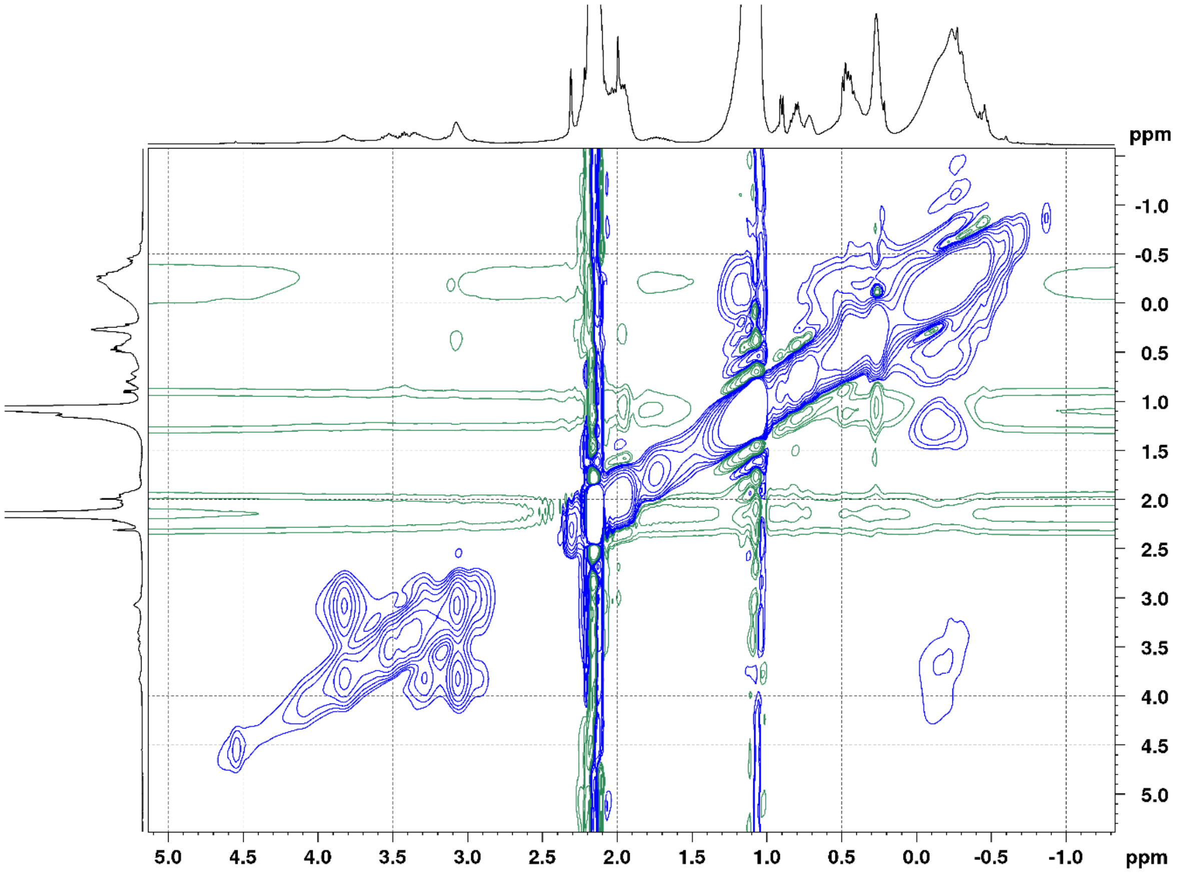

Figure S13. ^{1}H NMR of system $Me_2CCp_2ZrCl_2$ (**1c**) − $HAlBu^i_2$ − MMAO-12 (1:8:11) in C_7D_8 (298 K).

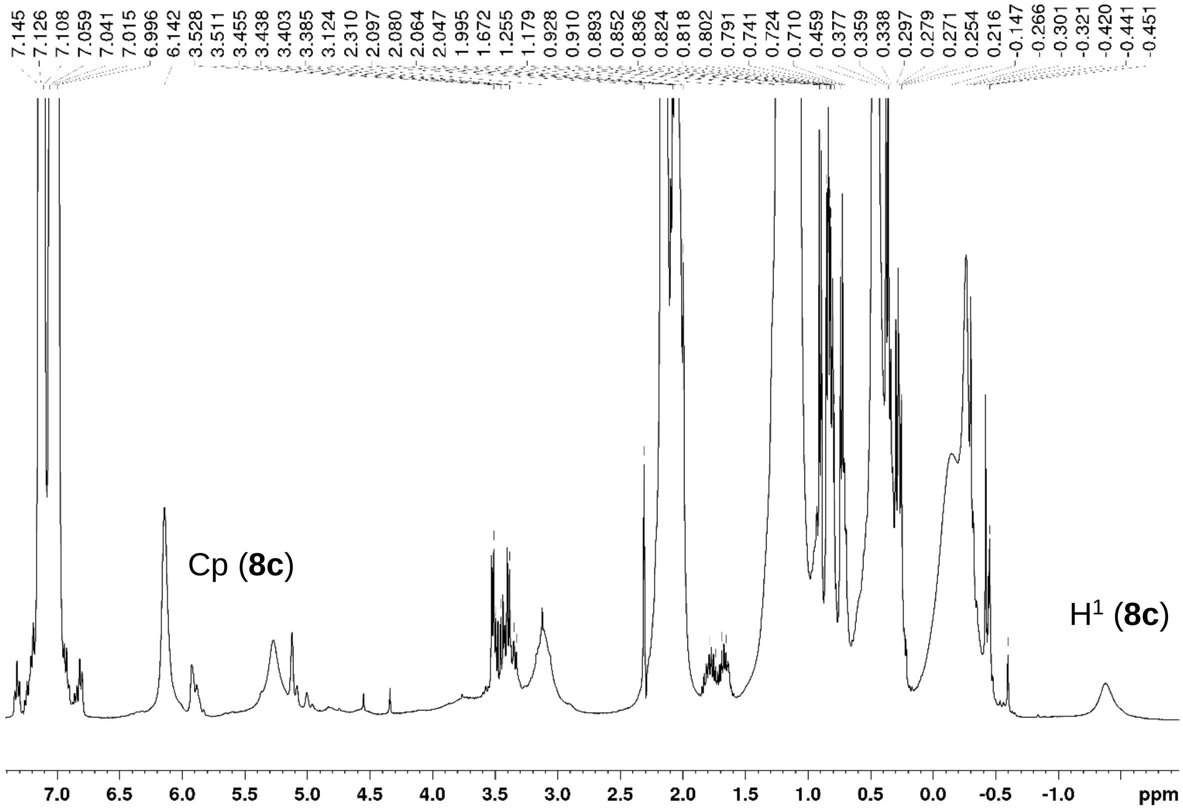

Figure S14. NOESY of system $Me_2CCp_2ZrCl_2$ (**1c**) − $HAlBu^i_2$ − MMAO-12 (1:8:11) in C_7D_8 (298 K).

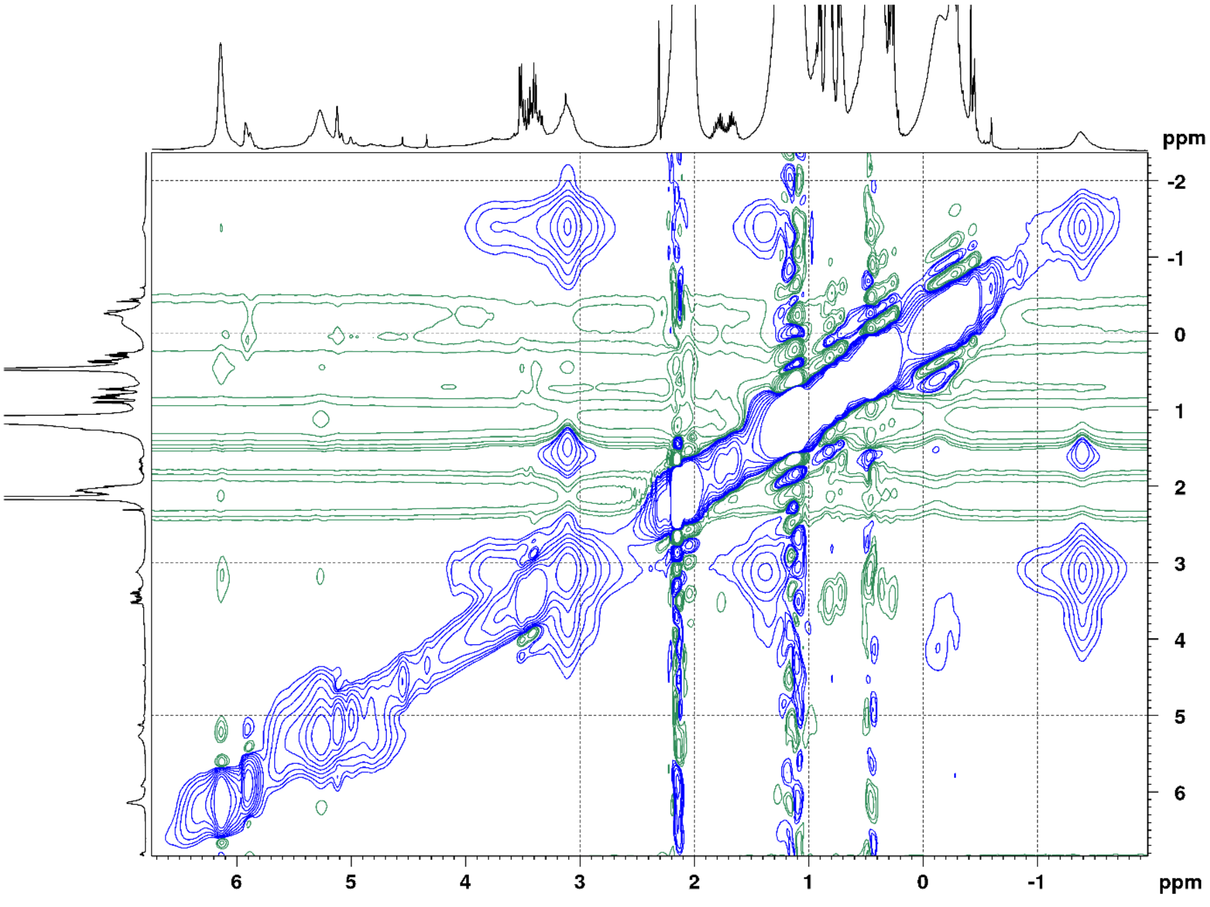

Figure S15. ^{1}H NMR of system $Me_2CCp_2ZrCl_2$ (**1c**) − $HAlBu^i{}_2$ – MMAO-12 (1:1.7:7) in C_7D_8 (298 K).

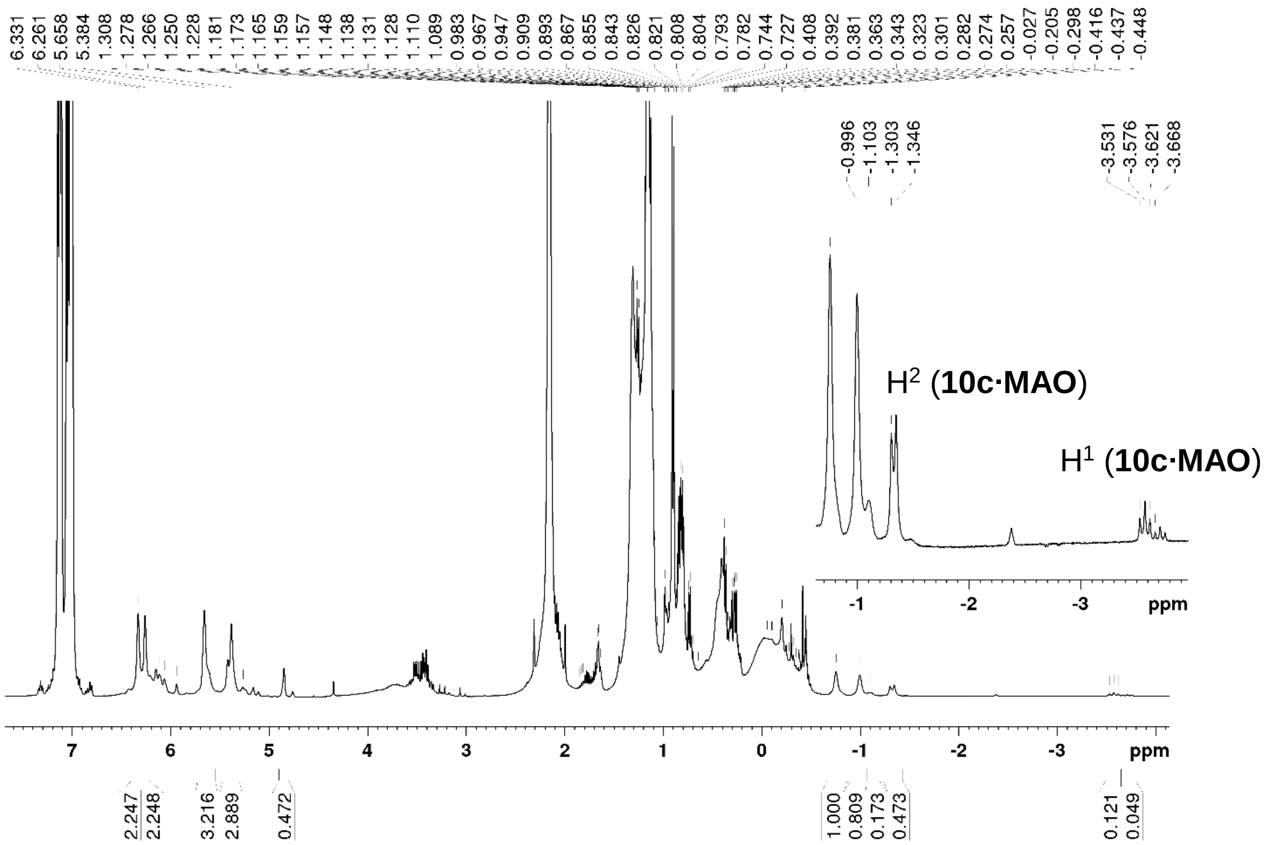

Figure S16. COSY HH of system $Me_2CCp_2ZrCl_2$ (**1c**) − $HAlBu^i{}_2$ – MMAO-12 (1:1.7:7) in C_7D_8 (298 K).

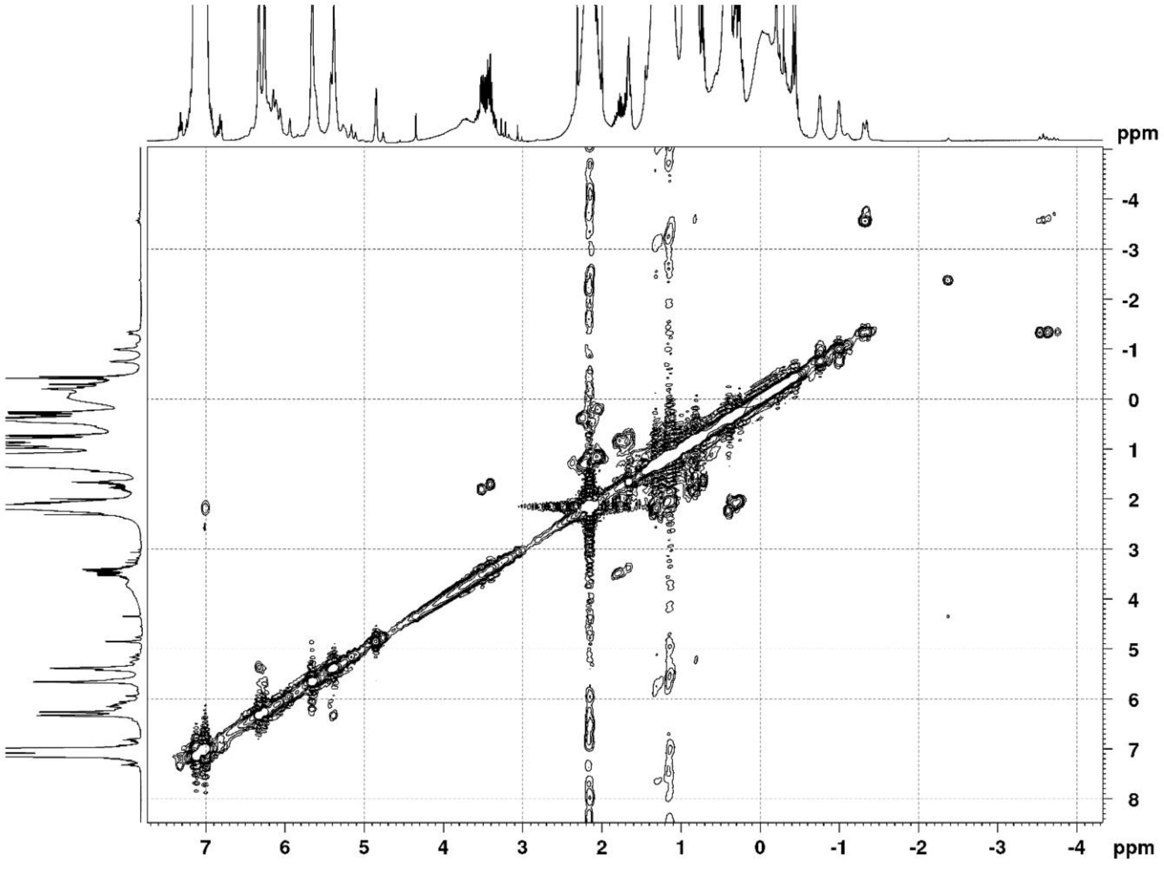

Figure S17. NOESY of system $Me_2CCp_2ZrCl_2$ (**1c**) − $HAlBu^i_2$ – MMAO-12 (1:1.7:7) in C_7D_8 (298 K).

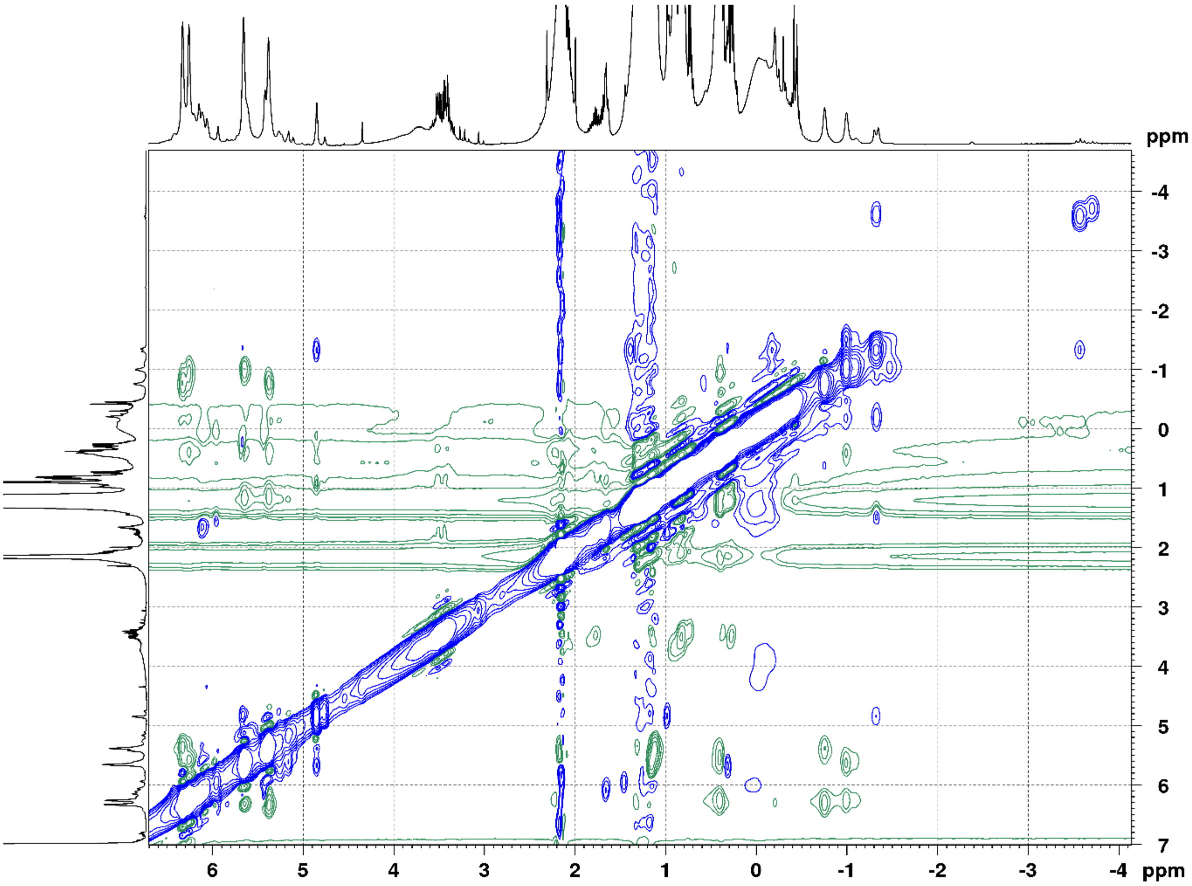

Figure S18. ^{1}H NMR of system $Me_2CCp_2ZrCl_2$ (**1c**) − $HAlBu^i_2$ – $(Ph_3C)[B(C_6F_5)_4]$ (1:1:0.1) in C_7D_8 (298 K).

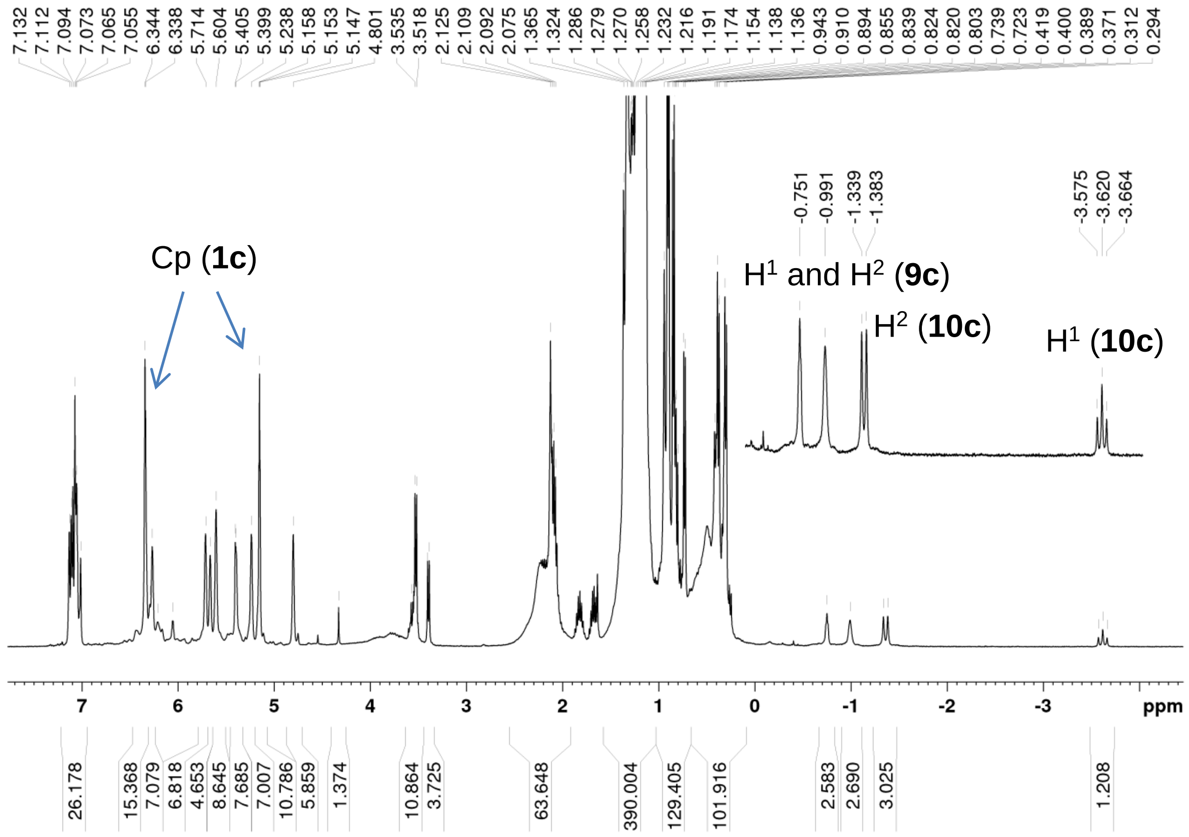

Figure S19. COSY HH of system $Me_2CCp_2ZrCl_2$ (**1c**) − $HAlBu^i_2$ – $(Ph_3C)[B(C_6F_5)_4]$ (1:1:0.1) in C_7D_8 (298 K).

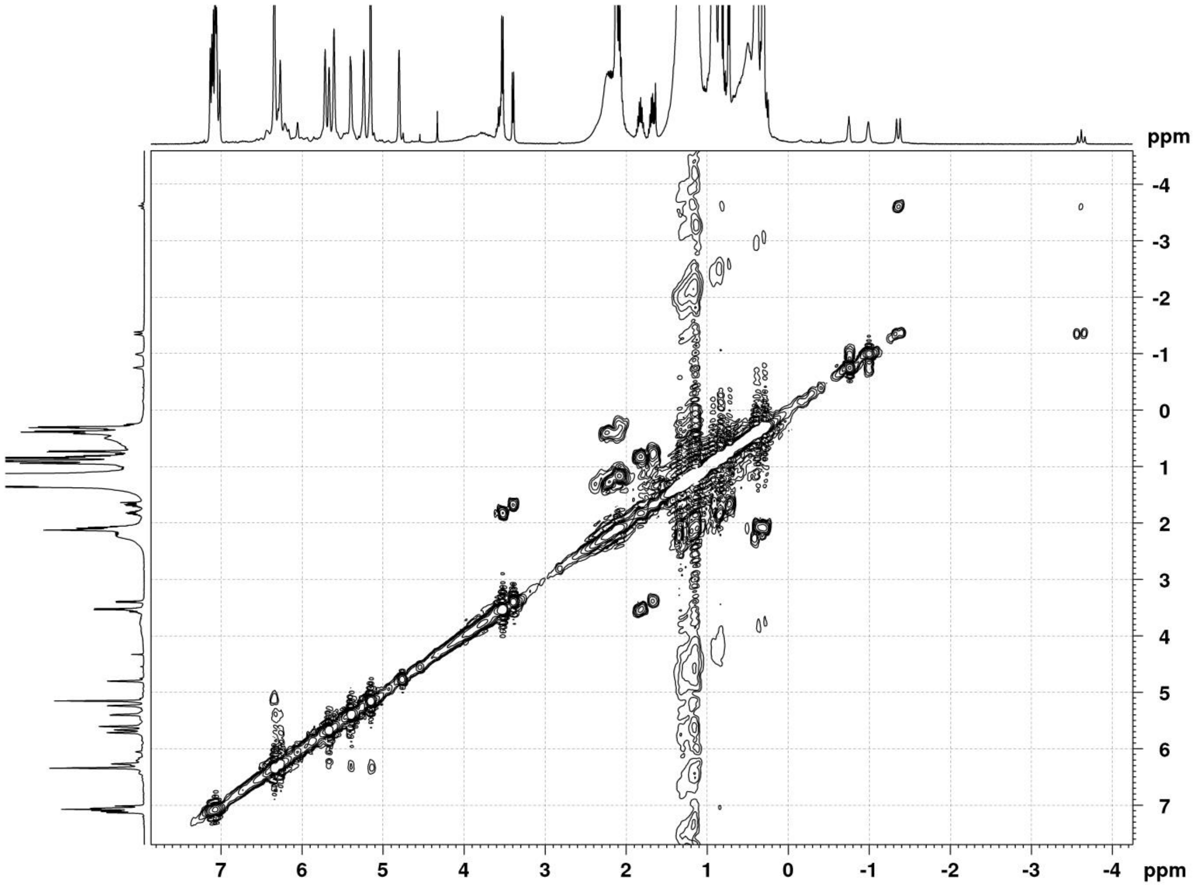

Figure S20. NMR monitoring of system $Me_2CCp_2ZrCl_2$ (**1c**) − $HAlBu^i_2$ − MMAO-12 – 1-hexene (1:8:11:2) in C_7D_8.

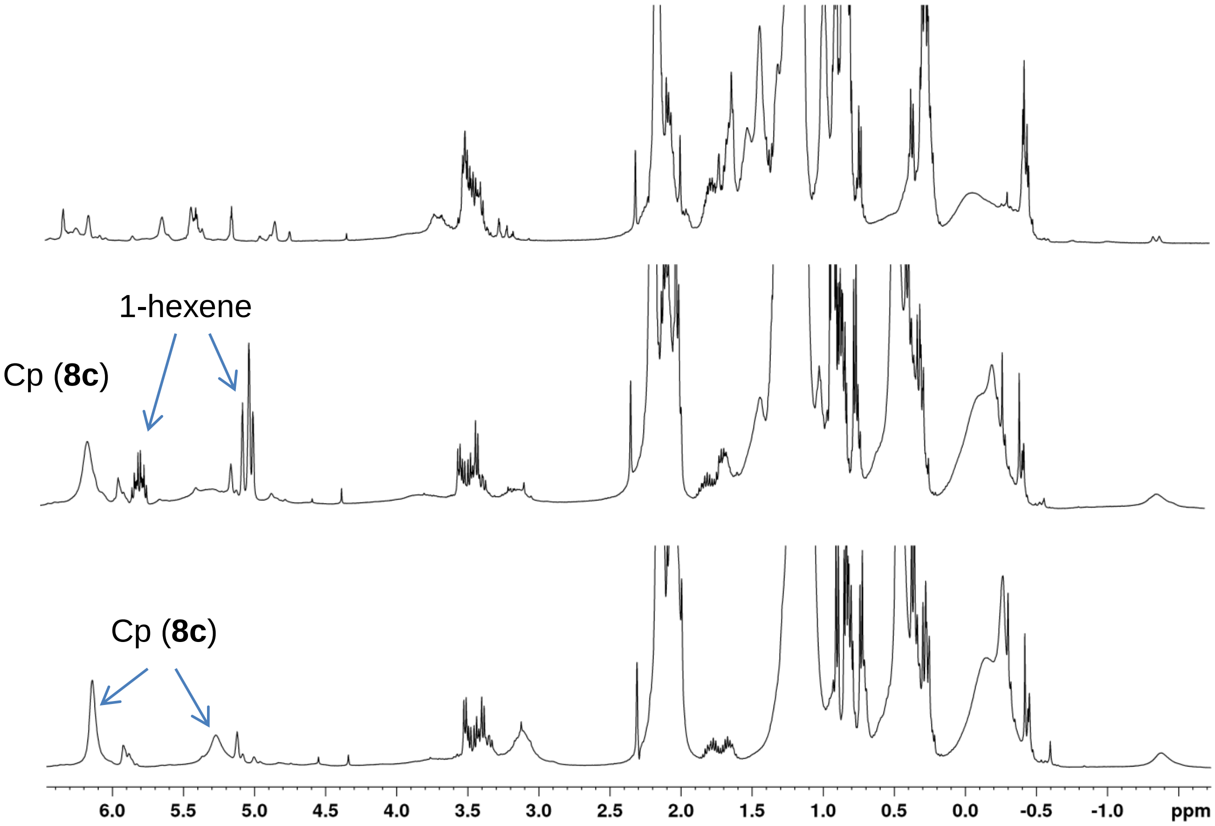

Figure S21. ^{1}H NMR of system Ind_2ZrCl_2 (**1f**) − $HAlBu^i_2$ (1:8) in C_7D_8: a) 220 K [43], b) 298 K.

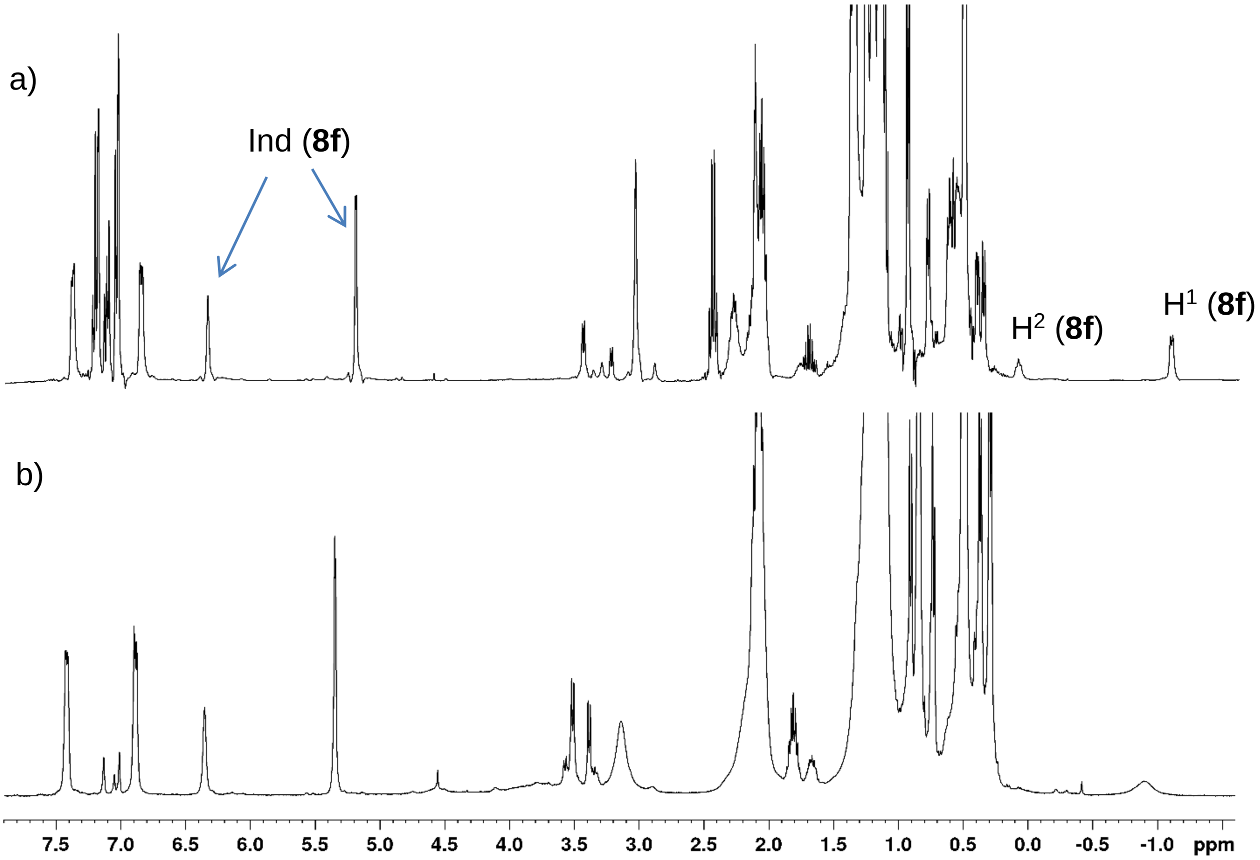

Figure S22. NOESY of system Ind_2ZrCl_2 (**1f**) − $HAlBu^i_2$ (1:3) in C_7D_8 (298 K).

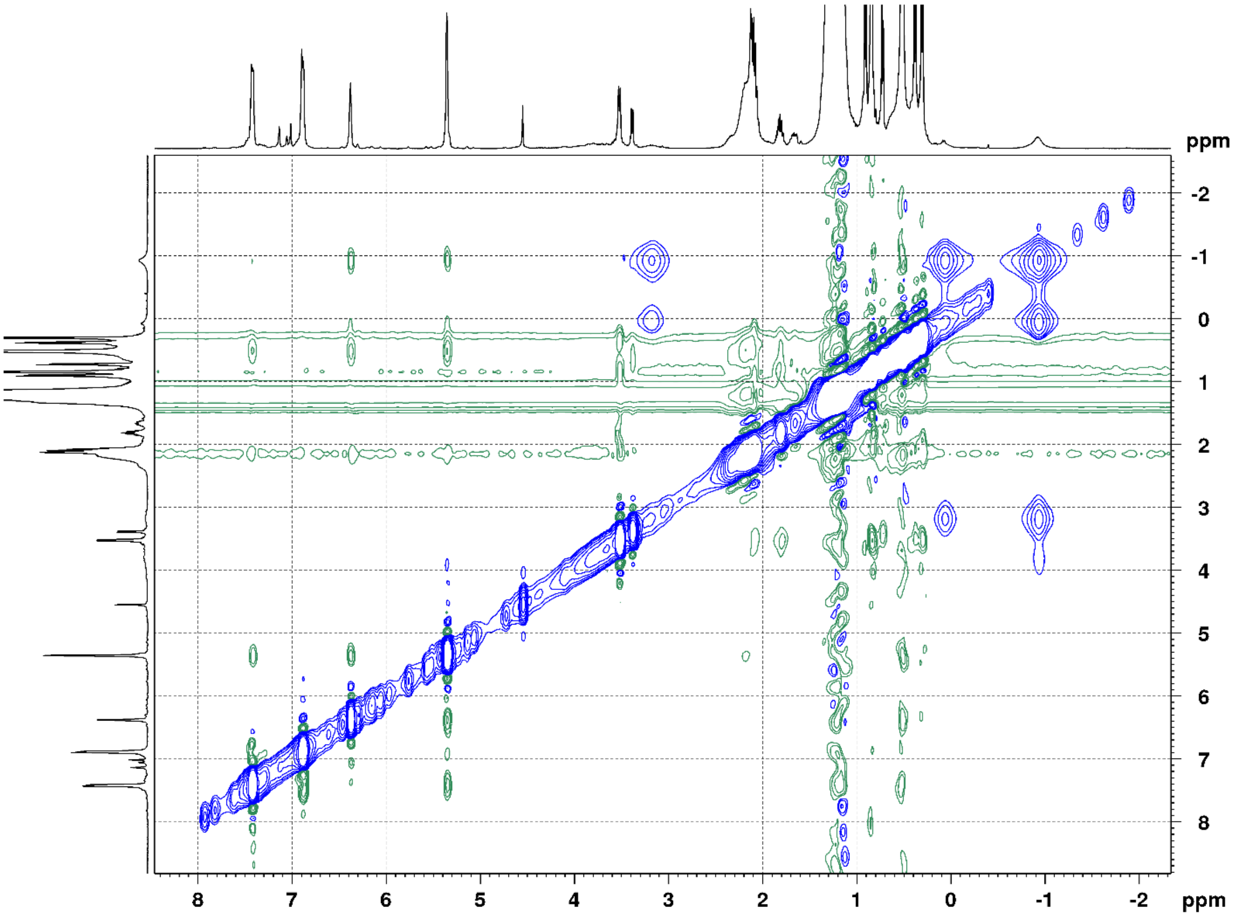

Figure S23. ^{1}H NMR of system Ind_2ZrCl_2 (**1f**) − $HAlBu^i_2$ – $(Ph_3C)[B(C_6F_5)_4]$ (1:3:0.2) in C_7D_8 (298 K).

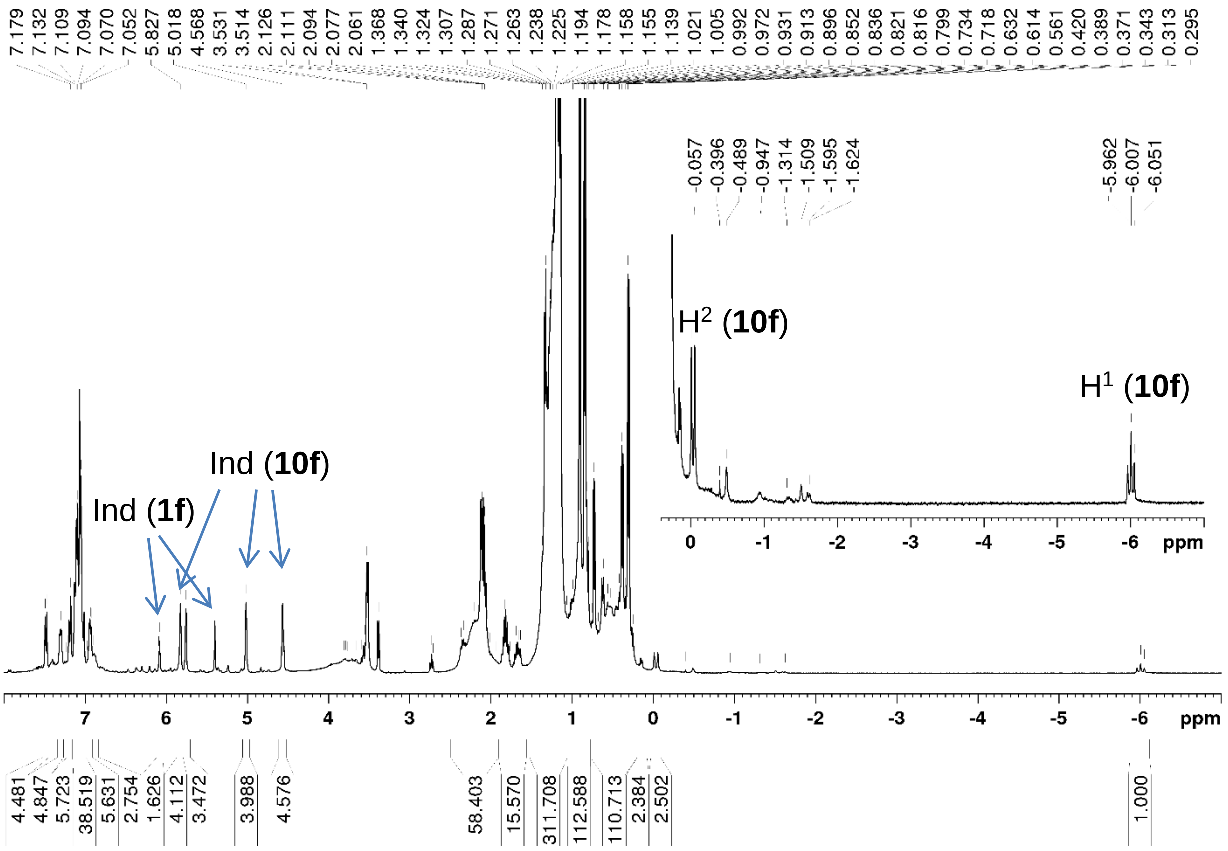

Figure S24. COSY HH of system Ind_2ZrCl_2 (**1f**) − $HAlBu^i_2$ – $(Ph_3C)[B(C_6F_5)_4]$ (1:3:0.2) in C_7D_8 (298 K).

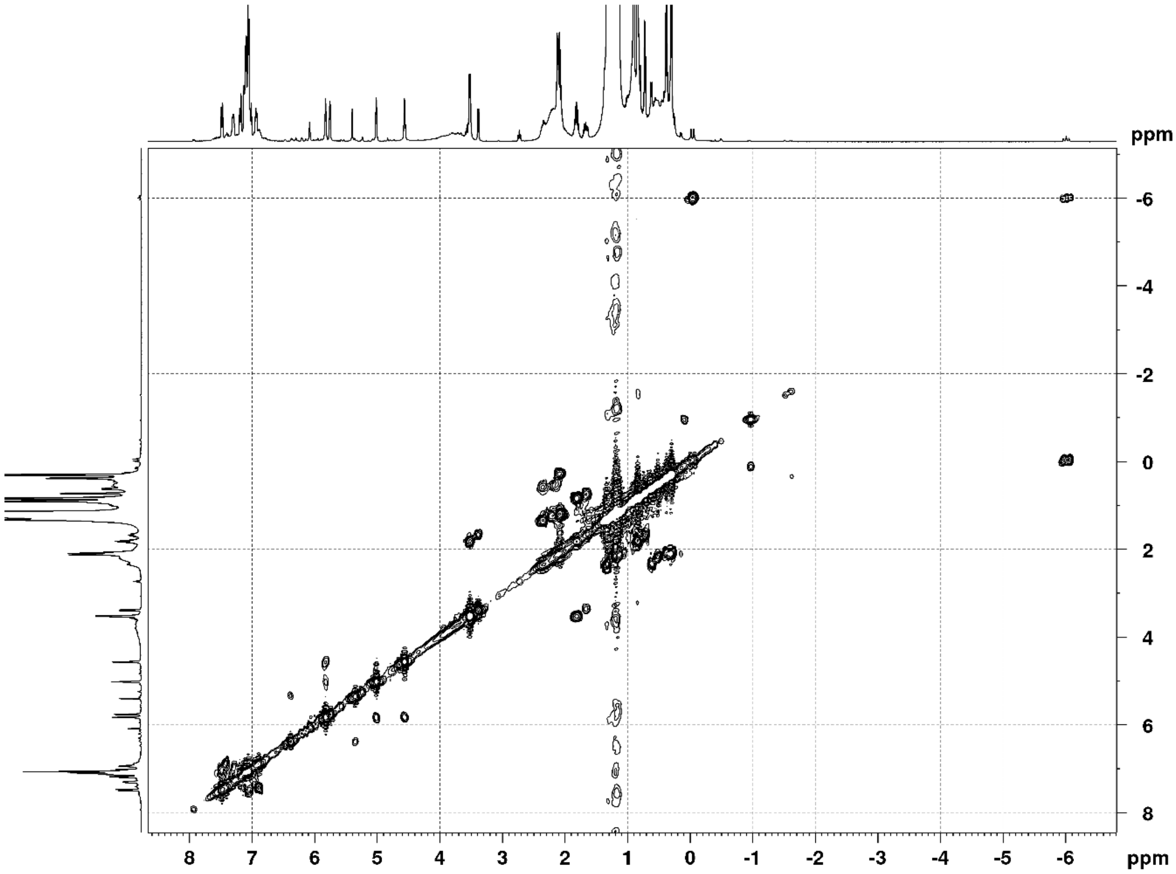

Figure S25. ^{1}H NMR of system Ind_2ZrCl_2 (**1f**) − $HAlBu^{i}_2$ – $(Ph_3C)[B(C_6F_5)_4]$ (1:8:0.2) in C_7D_8 (298 K).

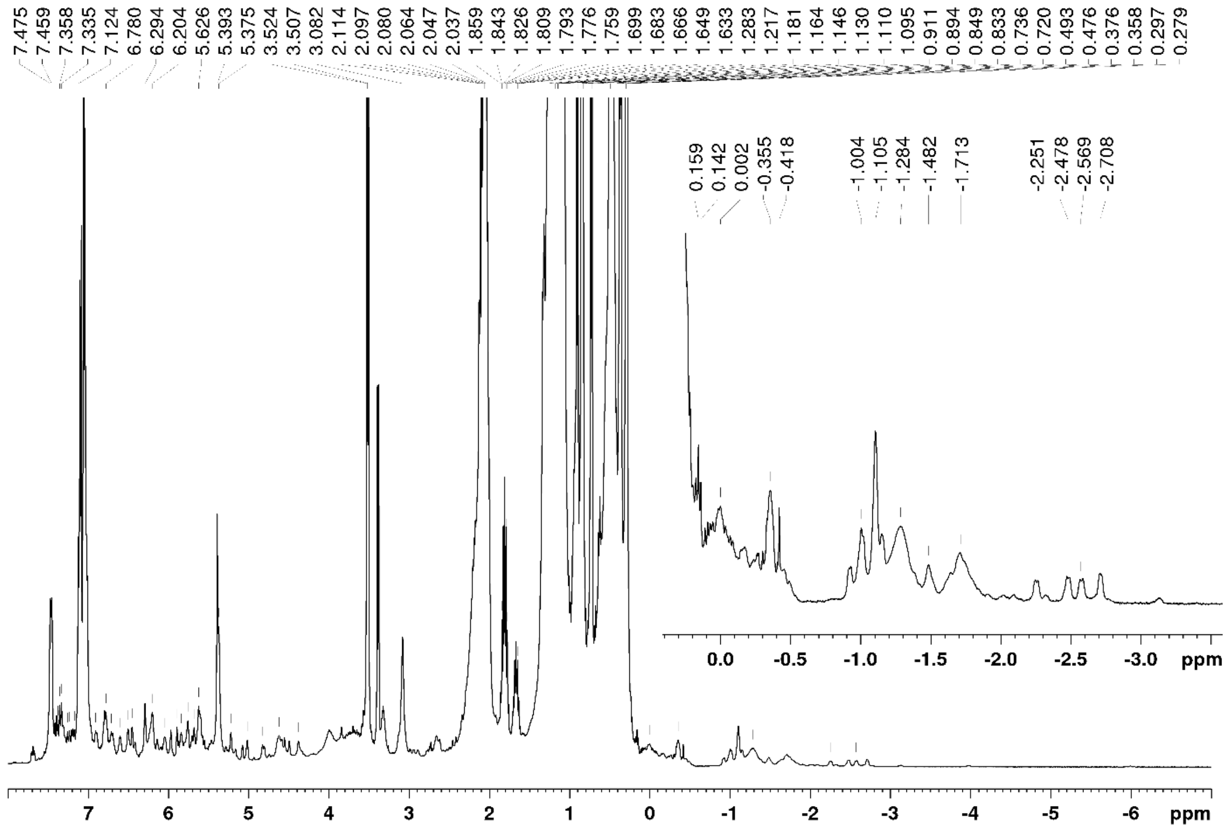

Figure S26. ^{1}H NMR of system Ind_2ZrCl_2 (**1f**) − $HAlBu^{i}_2$ – MMAO-12 (1:3:7) in C_7D_8 (298 K).

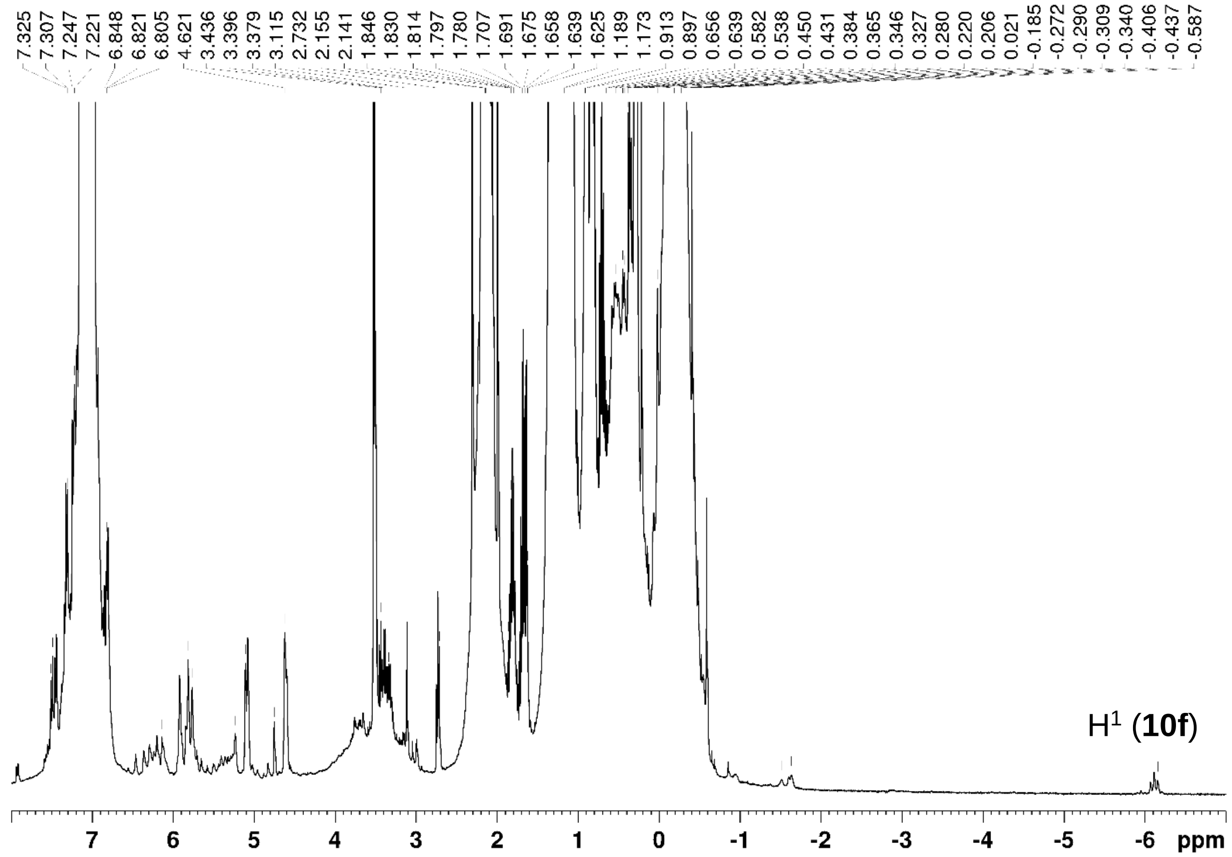

Figure S27. COSY HH of system Ind_2ZrCl_2 (**1f**) − $HAlBu^i_2$ – MMAO-12 (1:3:7) in C_7D_8 (298 K).

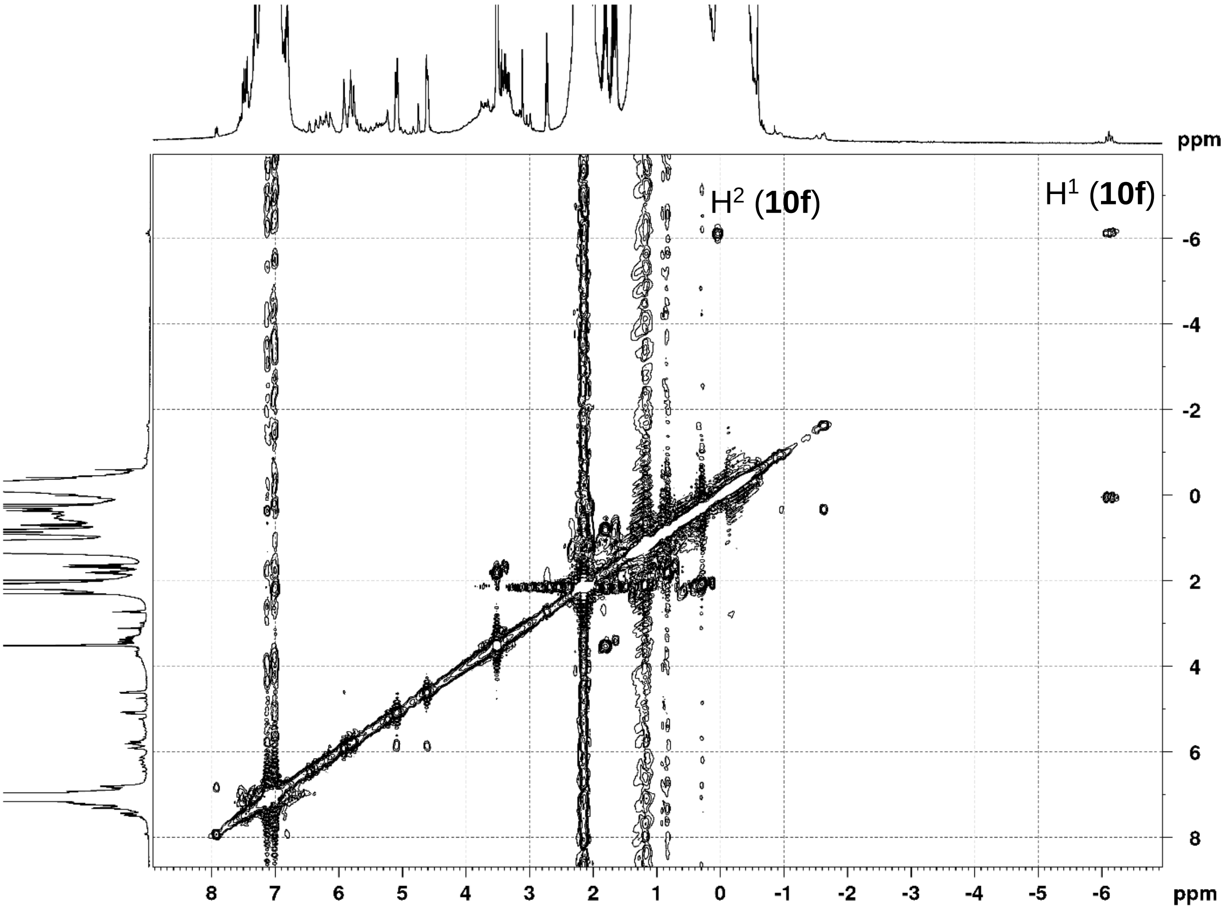

Figure S28. ^{1}H NMR of system Ind_2ZrCl_2 (**1f**) − $HAlBu^i_2$ – MMAO-12 (1:7:15) in C_7D_8 (298 K).

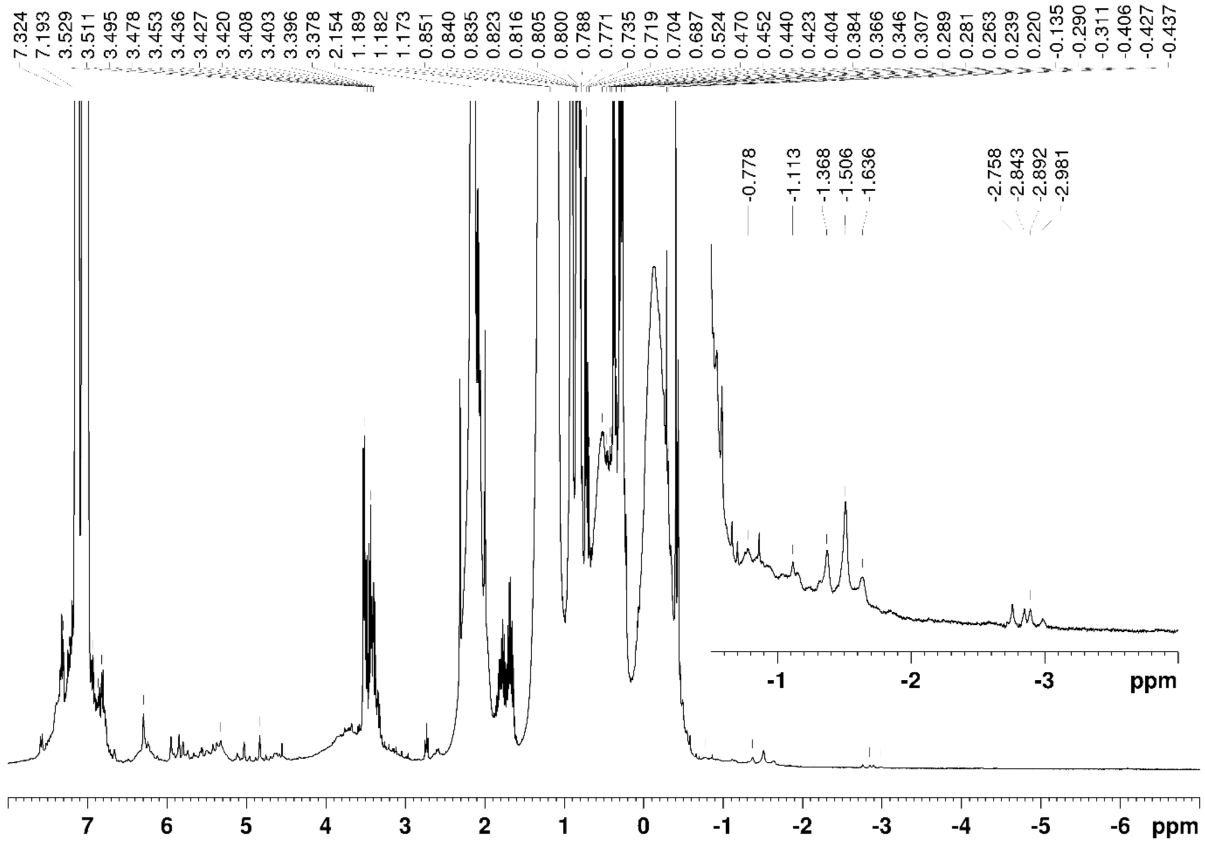

Figure S29. ^{13}C NMR of products obtained in the system Ind_2ZrCl_2 (**1f**) − $HAlBu^i_2$ − $(Ph_3C)[B(C_6F_5)_4]$ – 1-hexene in C_7D_8 at 298 K: a) [Zr]:[$HAlBu^i_2$]:[B]:[1-hexene]=1:3:0.2:5, 10 min; b) [Zr]:[$HAlBu^i_2$]:[B]:[1-hexene]=1:8:0.2:80, 10 min.

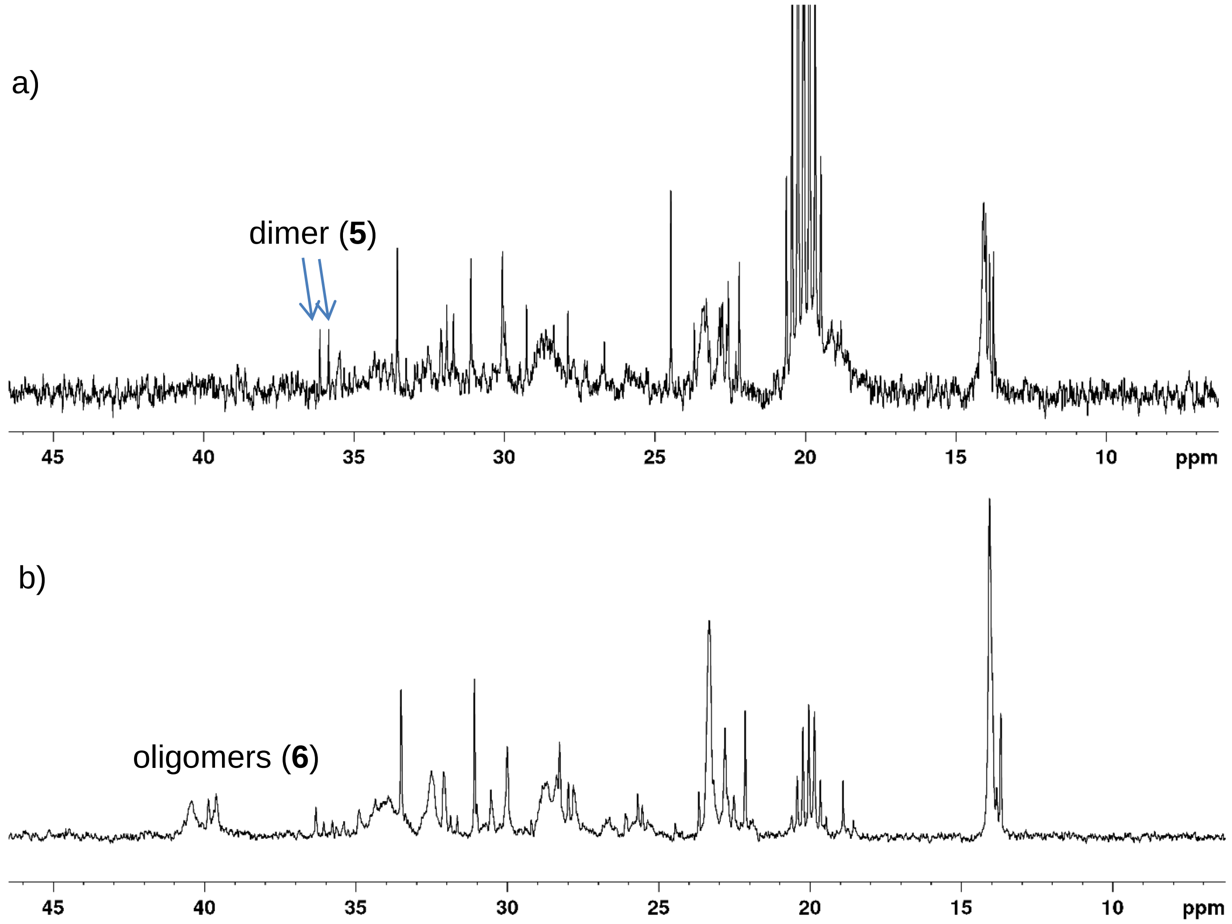

Figure S30. ^{13}C NMR of products obtained in the system Ind_2ZrCl_2 (**1f**) − $HAlBu^i_2$ – MMAO-12 – 1-hexene in C_7D_8 at 298 K: a) [Zr]:[$HAlBu^i_2$]:[Al_{MAO}]:[1-hexene]=1:3:7:3, 10 min; b) [Zr]:[$HAlBu^i_2$]:[Al_{MAO}]:[1-hexene]=1:8:10:80, 10 min.

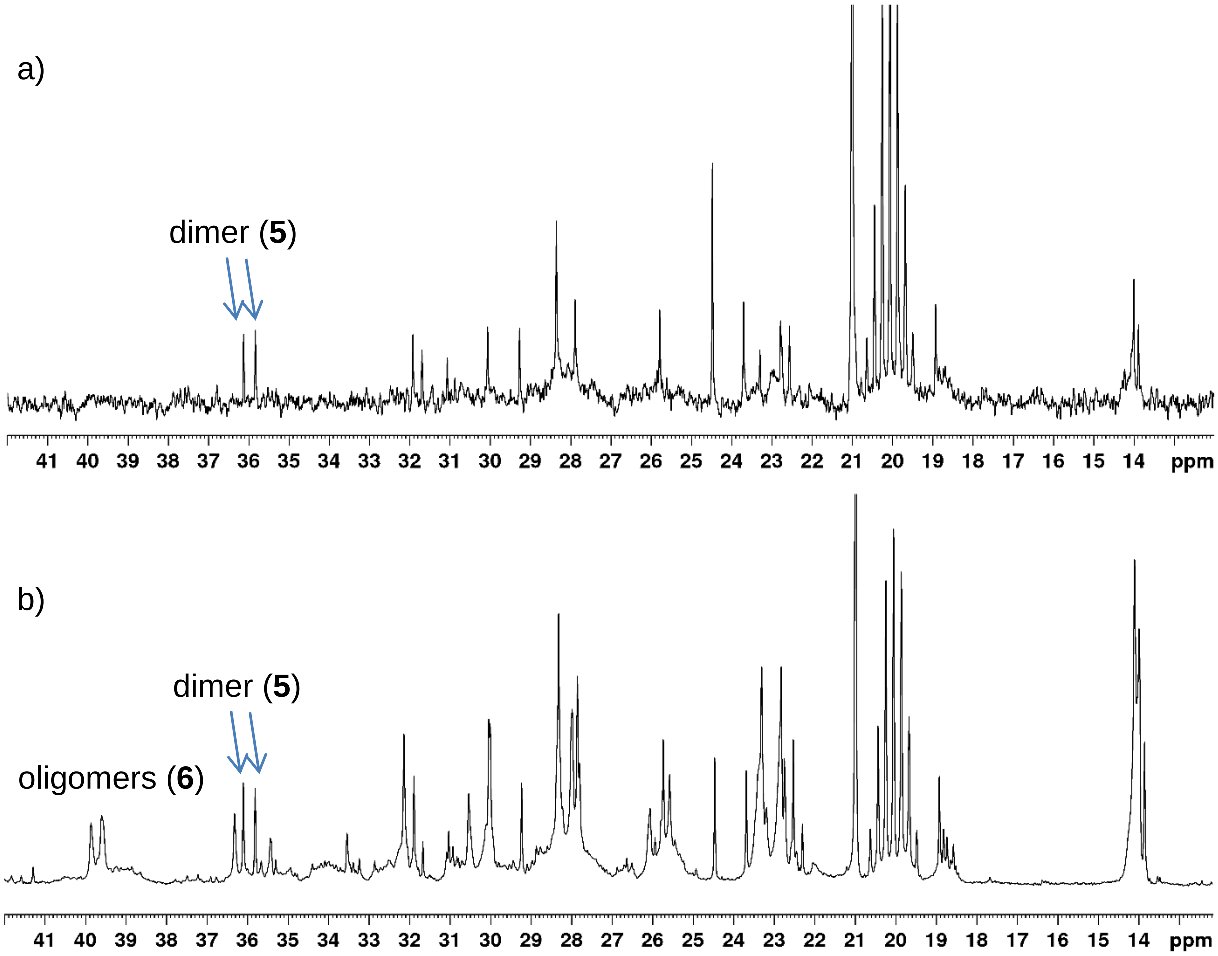

Supplement: Supplementary file 1 [file molecules-28-02420-s001.zip › molecules-2234625-supplementary.pdf]
